# Single and Combined Fe and S Deficiency Differentially Modulate Root Exudate Composition in Tomato: A Double Strategy for Fe Acquisition?

**DOI:** 10.3390/ijms21114038

**Published:** 2020-06-05

**Authors:** Stefania Astolfi, Youry Pii, Tanja Mimmo, Luigi Lucini, Maria B. Miras-Moreno, Eleonora Coppa, Simona Violino, Silvia Celletti, Stefano Cesco

**Affiliations:** 1 Department of Agricultural and Forestry Sciences, University of Tuscia, 01100 Viterbo, Italy; eleonoracoppa@libero.it (E.C.); simonaviolino@hotmail.com (S.V.); Silvia.Celletti@unibz.it (S.C.); 2 Faculty of Science and Technology, Free University of Bozen-Bolzano, 39100 Bolzano, Italy; youry.Pii@unibz.it (Y.P.); TMimmo@unibz.it (T.M.); stefano.cesco@unibz.it (S.C.); 3 Department for Sustainable Food Process, Università Cattolica del Sacro Cuore, 29122 Piacenza, Italy; luigi.lucini@unicatt.it (L.L.); mariabegona.mirasmoreno@unicatt.it (M.B.M.-M.)

**Keywords:** metabolomics, mugineic acid, iron, nutrient deficiency, nutrient interaction, phytosiderophores, root exudates, sulfur, strategy I, strategy II

## Abstract

Fe chlorosis is considered as one of the major constraints on crop growth and yield worldwide, being particularly worse when associated with S shortage, due to the tight link between Fe and S. Plant adaptation to inadequate nutrient availabilities often relies on the release of root exudates that enhance nutrients, mobilization from soil colloids and favour their uptake by roots. This work aims at characterizing the exudomic profile of hydroponically grown tomato plants subjected to either single or combined Fe and S deficiency, as well as at shedding light on the regulation mechanisms underlying Fe and S acquisition processes by plants. Root exudates have been analysed by untargeted metabolomics, through liquid chromatography–mass spectrometry as well as gas chromatography–mass spectrometry following derivatization. More than 200 metabolites could be putatively annotated. Venn diagrams show that 23%, 10% and 21% of differential metabolites are distinctively modulated by single Fe deficiency, single S deficiency or combined Fe–S deficiency, respectively. Interestingly, for the first time, a mugineic acid derivative is detected in dicot plants root exudates. The results seem to support the hypothesis of the co-existence of the two Fe acquisition strategies in tomato plants.

## 1. Introduction

Nowadays, agriculture is facing the dramatic challenge to increase the food production for an ever-growing world population, yet in a context of ever decreasing arable lands availability (also as a consequence of climate change). In this scenario, the use of marginal agricultural lands, characterized by unfavourable soil mineral composition, nutrients unbalance, water scarcity, acidity, salinization, etc. [1], could be particularly strategic. In this respect, it should be highlighted that nutrients availability is often one of the major limitations to the achievement of the best crop yield; unfortunately, this phenomenon is often also evident in the so-called fertile soils [2]. In these limiting conditions, the agronomical practice of fertilization is essentially the only usable strategy to cope with the problem. However, the decline in the availability of the natural resources at the base of the fertilizers production [3] is increasingly worrying from a long-term point of view. This context is even more complicated by the growing global demand for fertilizers due to the agricultural progress of the developing countries [4]. Considering all these aspects, it is possible to understand that current agricultural practices might be unsustainable for the future. For these reasons, the possibility of cultivating plant species that are particularly efficient in the exploitation of the nutritional resources already present in the soils appears particularly strategic and urgent. In fact, the reduction in both the environmental [5] and the economic [4] impact of agriculture, related to a massive use of fertilizers, appears to be one of the priorities. However, in order to overcome these issues, the understanding of the mechanisms underlying the acquisition processes of each single nutrient by roots surely plays a pivotal role. In this context, it should be also noted that, in this last decade, an interconnection between nutrients affecting the whole mineral nutrition process in plants has been clearly demonstrated [6]. In particular, the lack (deficiency) or excessive availability (toxicity) of a single nutrient is typically coordinated with the demand levels of other nutrients [7,8,9,10]. As a consequence, it might be postulated that the use efficiency of a single nutrient does not depend exclusively on its own availability in the growth medium. These aspects are particularly crucial for the efficacy of fertilization plans at the field scale. Therefore, the adoption of an approach that considers the global availability of nutrients becomes essential for a more complete understanding of mineral nutrition processes in crops, as well as for the exploitation of this agronomic practice in the context of a smart and more sustainable agriculture. Moreover, the identification/characterization of the sensing and signalling pathways activated in crops (as a consequence of the nutrients availability fluctuations is equally fundamental. In this respect, the mutual interaction between Fe and S has been clearly demonstrated in a wide variety of crops [11,12,13,14,15] and it was shown to have impacts at both the acquisition and assimilation levels. Regarding the latter, it is worth mentioning the Fe biofortification of wheat grain achieved through overfertilization with sulfate [16]. In this context, the recent environmental policies that have led to a reduction in S-rich industrial emissions, therefore decreasing the free S deposition at the soil with a consequent and progressive S depletion in the arable soil [17], have aroused even more interest in this mutual Fe/S interaction. The expected further decrease in soil S contents in the future will further contribute to stimulating interest in this regard. This aspect is even more intriguing if we consider that, differently from S, Fe is abundantly present in soils, although its available fraction is very often insufficient to guarantee a balanced growth of the crop [18,19].

Plant adaptation to an environment characterized by an inadequate nutrient availability is a process relying on several often sophisticated strategies aimed at enhancing the nutrients mobilization from soil minerals and at favouring their acquisition by roots. These strategies also include the release of root exudates (a mixture of organic acids, phenolic compounds, sugars, vitamins, amino acids, inorganic molecules, enzymes, and root border cells, [19,20] directly involved in the biogeochemical cycles of nutrients in soils [21]. Once synthesized inside the plant cells, they are then released by the roots into the rhizosphere through diversified root exudation mechanisms [22,23]. From the agronomic point of view, an example of their function is represented by the root secretion of extracellular enzymes that, via the *mineralization* process within the organic matter cycle, can significantly increases the rhizosphere availability of nutrients like N, P, S and metals previously preserved in the organic matrix [23]. Differently, based on the different solubility of nutrients in soil minerals, the acidification or alkalinisation of the rhizosphere as a consequence of the root release of exudates like H^+^, HCO_3_^−^, organic acids, etc., can considerably favour or hinder the mobilization process of different nutrients/elements, such as P, K, Fe, Zn, etc. [24,25]. In this respect, the enhanced exudation of organic ligands like phytosiderophores (PS) and organic acids with a high affinity for elements/metals (such as Fe) can also have a very positive impact on the metal mobilization (mineral weathering) [19,26,27,28]. With respect to the organic acids, it should be mentioned that they can also impact the rhizosphere processes and the fertility levels of this soil volume through their direct effect on the microbial biomass and its activity [29,30].

In particular, with respect to Fe, plants have developed two mechanisms, named Strategy I and Strategy II, aimed at coping with this nutritional disorder [31]. The Strategy I mechanism starts by inducing H^+^-ATPase activity to solubilize Fe^3+^, which is then reduced to Fe^2+^ by the increased activity of a membrane-bound Fe^3+^-chelate reductase and the resulting Fe^2+^ is transported into the plant root cell by a Fe^2+^-transporter [31]. Strategy II plants (only grasses) increase the synthesis and release into the rhizosphere of ligands with a high affinity for Fe, named PS, concomitantly with an enhanced uptake of FeIII-PS complexes via a specific transporter (YellowStripe1, YS1) [31].

It has been clearly shown that root exudates are involved in both adaptative mechanisms (FeIII-reduction-based *Strategy I* of dicots and FeIII-PS complexes-based *Strategy II* of grasses) developed by plants in order to guarantee the Fe homeostasis in the different tissues [31] and some studies have shown that the composition of root exudates can change significantly as a function of the diverse levels of Fe availability [32,33,34,35]. However, despite the mutual interaction between Fe and S being well documented for the uptake mechanisms of the respective nutrients [11,12,13,14,15], to our knowledge there are still no indications regarding the role of this phenomenon in the exudate composition. From a general point of view, it is reasonable to hypothesize an effect of the variable S availability on the exudative pattern of roots with consequent adjustments to the organic compound fluxes in the soil. This aspect is particularly relevant considering that the potential capacity of the roots to modify effectively the rhizosphere to their own advantage depends on the quali-quantitative composition of exudates [20,22,23]. Moreover, considering that, at the field level, the onset of S deficiency symptoms is not only increasingly likely in the future but also, when present at a greater gravity extent, it appears evident how crucial the characterization and comprehension of this phenomenon is. 

Therefore, on the basis of these premises, in the present work the root exudation pattern as well as the chemical composition of these exudates have been evaluated using tomato plants exposed to different levels of nutrient availability, i.e., single Fe, single S, or combined Fe–S deficiency. Results suggest that the pool of metabolites released in the rhizosphere, belonging to the primary and secondary metabolism, is considerably adapted to the diverse external nutrient conditions, i.e., the single deficiency of Fe or S, or the combination of both Fe–S deficiencies.

## 2. Results

### 2.1. Plant Growth Features

**Figure 1:**
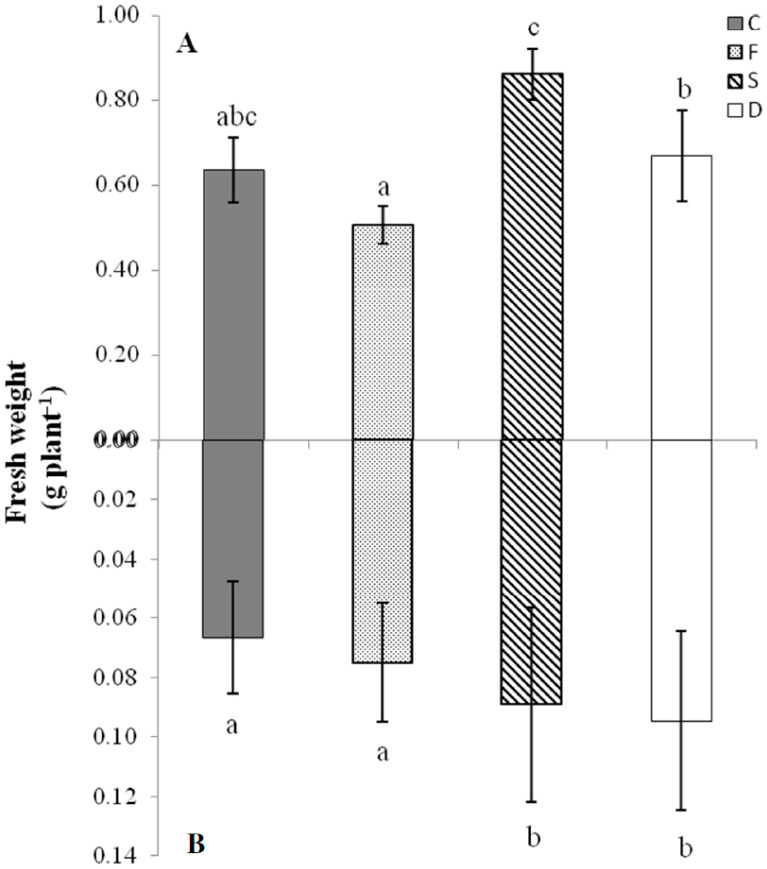
Biomass accumulation of tomato plants. Shoot (**A**) and root (**B**) fresh weight (g plant^−1^) of tomato plants subjected to different nutritional treatments (C = control, F = Fe deficiency, S = S deficiency, D = dual deficiency). Data are means ± SD of six independent replicates run in triplicate. The statistical significance has been tested by a one-way ANOVA test with Tukey post-test. Significant differences between samples are indicated by different letters (*p* < 0.01).

**Figure 2:**
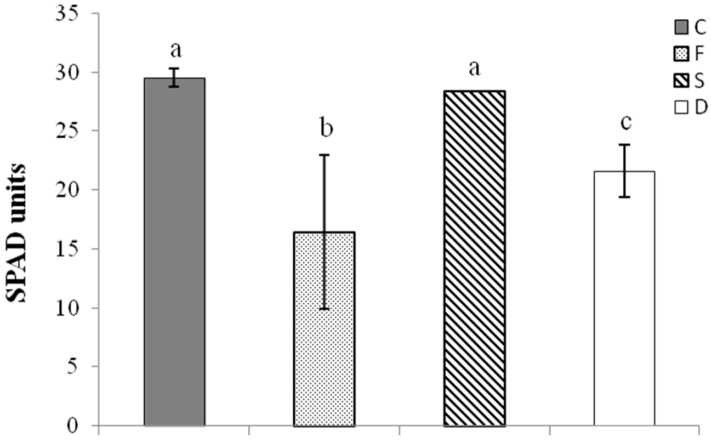
Chlorophyll content in tomato leaves. Changes in chlorophyll levels, evaluated as SPAD units, in leaves of tomato plants subjected to different nutritional treatments (C = control, F = Fe deficiency, S = S deficiency, D = dual deficiency). Data are means ± SD of six independent replicates run in triplicate. The statistical significance has been tested by a one-way ANOVA test with Tukey post-test. Significant differences between samples are indicated by different letters (*p* < 0.01).

All nutrient deficiency treatments significantly affected the growth features of tomato seedlings (Figure 1 and Figure 2). Single Fe deficiency (F) did not affect plant growth, but resulted in a strong decrease in chlorophyll content, estimated as SPAD units (−45%, with respect to control condition). Single S deficiency (D) resulted in a notable increase in root apparatus development (+33% with respect to control condition). On the other hand, the variables affected by combined Fe and S deficiency (D condition) included root growth, which was increased by 42% and chlorophyll content, which was reduced by 24%. The differences between treatments were further tested via multivariate analysis of variance (MANOVA) considering the shoot weight, root weight, and chlorophyll content. As shown in Supplementary Table S2, all the pairwise comparisons resulted to be significant, except for treatment C vs. S.

### 2.2. Metabolomic Profiling of Root Exudates

**Figure 3:**
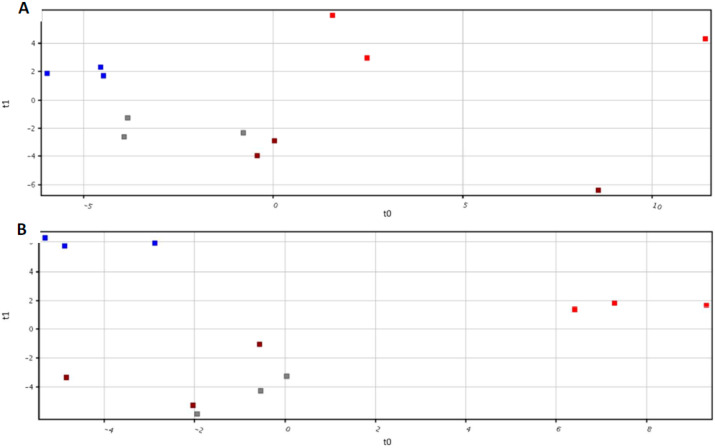
Partial Least Squares Discriminant (PLS-DA) multivariate modelling from the dataset obtained through GC-MS (**A**) and UHPLC/QTOF-MS (**B**) analysis in the root exudates of plants exposed to the different nutritional conditions (Grey = Control, Blue = Fe deficiency, Brown = S deficiency, Red = dual deficiency).

Root exudates of tomato plants were collected 9 days after the treatments, by submerging roots in a water-based trap solution, as previously described [34,36]. Root exudates were thereafter subjected to untargeted metabolomic analyses through complementary GC-MS and UHPLC/QTOF-MS approaches. The PLS-DA multivariate modelling carried on the dataset obtained through GC-MS technique highlighted four independent clusters (Figure 3A). A similar clustering pattern was also obtained from UHPLC/QTOF-MS profiles, even though in this case S and C conditions were partially overlapping (Figure 3B). PLS-DA accuracy, following *n*-fold validation (*n* = 3) was 100% for both GC-MS and UHPLC/QTOF-MS modelling.

**Figure 4:**
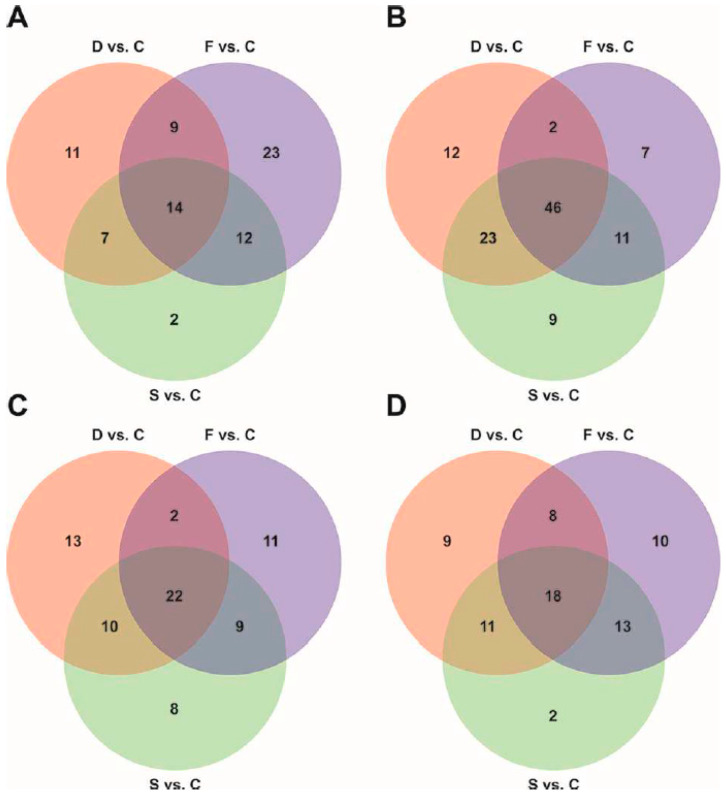
Venn diagrams representing the differentially represented metabolites detected in the root exudates of plants exposed to the different nutritional conditions. (**A**) Number of up-regulated metabolites detected by GC-MS techniques and normalized on control samples. (**B**) Number of down-regulated metabolites detected by GC-MS techniques and normalized on control samples. (**C**) Number of up-regulated metabolites detected by HPLC-MS techniques and normalized on control samples. (**D**) Number of down-regulated metabolites detected by HPLC-MS techniques and normalized on control samples.

The Volcano Plot statistical analysis carried out on GC-MS data highlighted 124, 124 and 124 differentially represented metabolites in the root exudates of F, S and D plants, respectively, as compared to C plants (Table S2). The complete list of metabolites is provided as supplementary material (Table S3). Within the metabolites differentially represented in the exudates of F plants, 58 were up-accumulated and 66 down-accumulated as compared to C plants (Table S2). In the case of S and D plants, the majority of the differentially regulated metabolites were down-accumulated (89 and 83, respectively), whilst only 35 and 41 were over-represented as compared to control plants (Table S2). Among the up-accumulated metabolites, 14 were released in all the physiological conditions, whereas 23, 2 and 11 were characteristic for F, S and D plants, respectively (Figure 4A). On the hand, 46 metabolites were commonly down-accumulated in all the stress conditions imposed, whilst the lack of 7, 9 and 12 metabolites was a distinctive feature of F, S and D plants, respectively (Figure 4B).

The UHPLC/QTOF-MS technique allowed the identification of 93, 93 and 83 differentially represented metabolites in the root exudates of F, S and D plants, respectively, as compared to C plants (Table S4). The profile of differentially represented metabolites in F and D conditions was similar; in fact, we observed 44 and 49 up-accumulated metabolites and 49 and 44 down-accumulated metabolites, as compared to control, in F and S plants, respectively). In the case of D condition, 47 and 36 metabolites were up- and down-accumulated, respectively. Within the group of up-accumulated metabolites, 22 were over-represented in all the physiological conditions with respect to control, whereas 11, 8 and 13 were characteristic for F, S and D plants, respectively (Figure 4C). On the contrary, 18 metabolites were commonly down-accumulated in all the nutritional conditions as compared to controls, whilst the reduction in 10, 2 and 9 metabolites was a distinctive feature of F, S and D plants, respectively (Figure 4B).

### 2.3. ChemRICH Set Enrichment Analysis

Due to the large dataset provided by the metabolomics approach, clustering metabolites is a method to obtain a deeper insight in the biological processes. In this study, encoded chemical structures were used to cluster and define the metabolites. Since some compounds have not yet been classified in chemical ontologies, chemical similarity for clustering the principal classes of metabolites is emerging as a powerful tool. 

**Figure 5:**
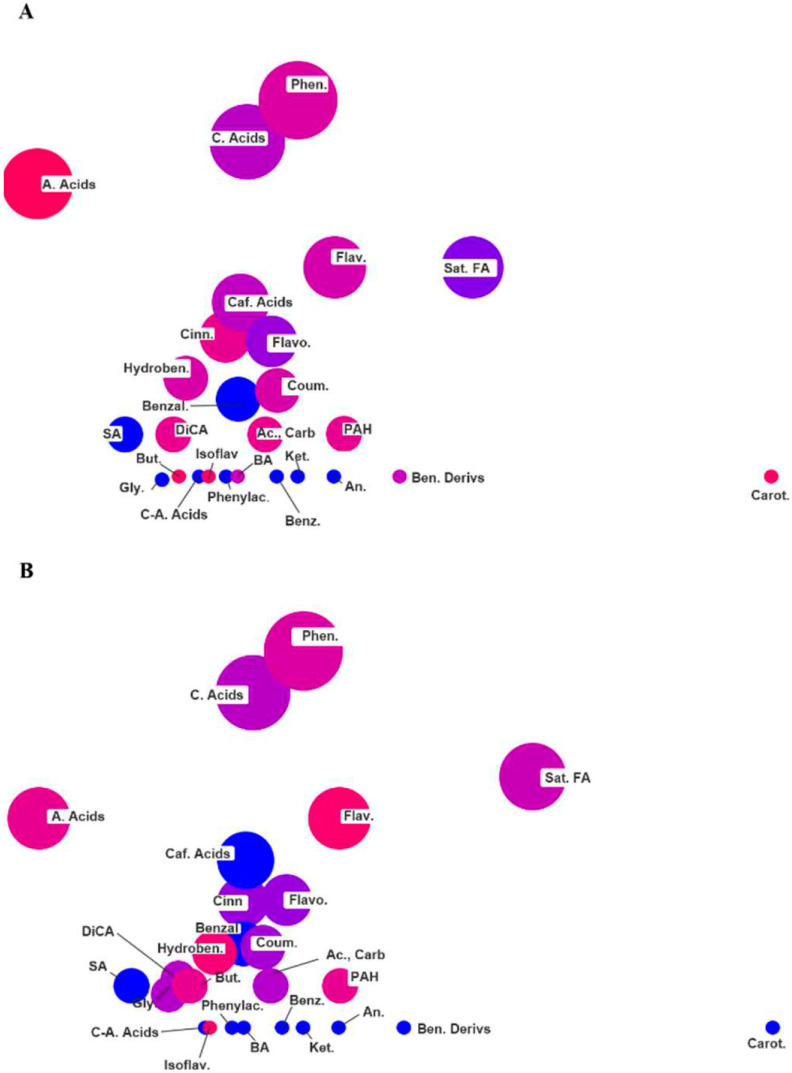

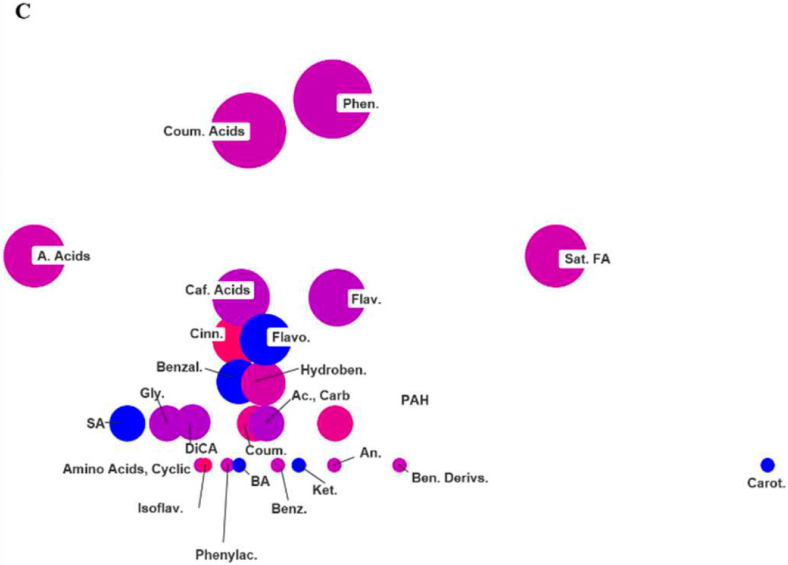
Chemical similarity enrichment analysis (ChemRICH diagrams) of the metabolites found in plants under Fe deficiency (**A**), S deficiency (**B**) and dual deficiency (**C**), as obtained by UHPLC/QTOF-MS and GC-MS. Color is according to proportion of increased or decreased compounds (red = increased, blue = decreased, pink = mixed) within each cluster. A. Acids: amino acids; Ac., Carb.: Acids, Carbocyclic; An.: anisoles; BA.: Benzyl Alcohols; Ben. Derivs: benzene derivatives; Benz.: benzoates; Benzal.: benzaldehydes; But.: butyrates; C.-A. Acids: Amino AcidsCyclic; Caf. Acids: caffeic acids; Carot.: carotenoids; Cinn.: cinnamates; Coum. Acids: coumaric acids; Coum.: coumarines; DiCA.: dicarboxylic acids; Flav.: flavonoids; Flavo.: Flavones; Gly.: glycols; Hydroben.: hydroxybenzoates; Isoflav.: Isoflavone; Ket.: Ketones; PAH.: Polycyclic Aromatic Hydrocarbons; Phen.: phenols; Phenylac.: phenylacetates; SA.: Sugar Alcohols; Sat. FA.: saturated fatty acids.

**Figure 6:**
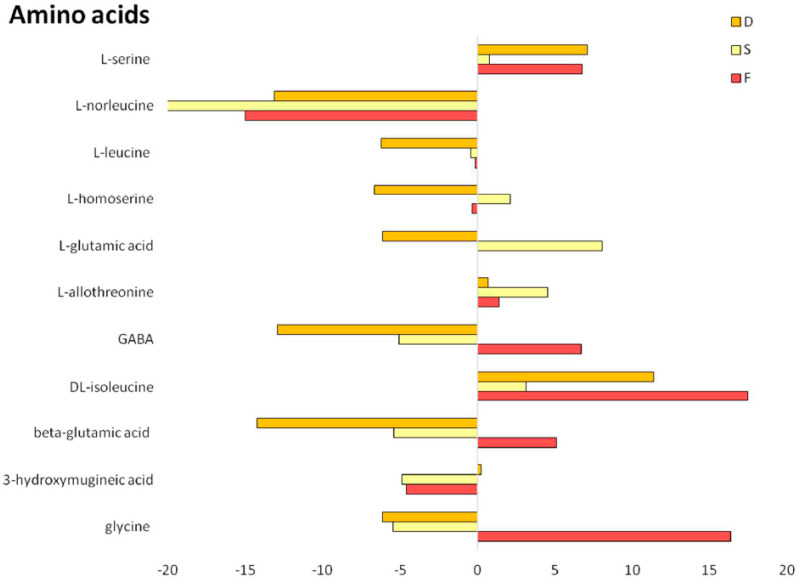
Changes in amino acids release induced by different nutritional regimes. Amino acids in root exudates from tomato plants subjected to different nutritional treatments (C = control, F = Fe deficiency, S = S deficiency, D = dual deficiency). The figure shows metabolites that change significantly in F, S or D condition with respect to control samples *p* < 0.001) and changes are expressed as fold changes (treatment/control ratio).

In this sense, Tanimoto substructure chemical similarity coefficients were used to cluster metabolites into non-overlapping chemical groups to better understand the complexity of the metabolite signatures [37]. ChemRICH set enrichment statistics plot identified different chemical clusters being phenols, coumaric acids and amino acids the most represented cluster in root exudates under all conditions (Figure 5). All the treatments showed a similar profile of characteristic metabolites (Table S5), in terms of abundance and statistical significance. Nevertheless, different accumulation patterns of the represented chemical classes could be described. In particular, flavones were over-represented in F and S plants (Figure 5A,B) and down-accumulated in the double deficiency (D) condition (Figure 5C), whereas carotenoids increased in the root exudates of Fe-deficient plants (F) and decreased in those of S-deficient and double-deficient plants (Figure 5). In the root exudates of F and D plants, an increase in the concentration of caffeic acids was also detected, while saturated fatty acids decreased in F condition and increased in S and D (Figure 5). Phenols, coumaric acids, flavonoids, amino acids, and coumarins were always up-accumulated in all the nutritional conditions considered (i.e., F, S and D) as compared to C conditions (Figure 5), albeit to a different extent. Interestingly, the secretion of coumarins by roots of D plants was more enhanced than under F and S conditions, whereas total free amino acid concentration increased in F and S plants compared to the D ones (Figure 5). In particular, 11 amino acids were identified in root exudates of tomato plants, and changes associated with single or combined Fe- and S-deficiency were detected (Figure 6). Up-accumulation was recorded for serine, allothreonine and isoleucine, whereas down-accumulation was observed for norleucine and leucine, irrespective of the nutritional treatment (Figure 6). The main change in plants grown in a Fe-deficient medium, relative to plants grown in S and D conditions, was the up-accumulation of the amino acids GABA, beta-glutamic acid and glycine (with increases of 6.7-, 5.1- and 16.3-folds than control, respectively) (Figure 6). On the other hand, the main change in plants grown in S-deficient medium, relative to plants grown in F and D conditions, was the up-accumulation of the amino acids homoserine and glutamic acid, with 2.1-fold and 8-fold increases as compared to control, respectively (Figure 6). Interestingly, in tomato root exudates, hydroxymugineic acid was found and, more importantly, it was found to be down-regulated under both F and S conditions, with 4-fold lower concentrations than control (Figure 6). Finally, changes associated with combined deficiency (D) followed F or S patterns, even if to a different extent, but the abundance of hydroxymugineic acid was similar to that of control (Figure 6).

### 2.4. Bioinformatics and Gene Expression Analysis

**Figure 7:**
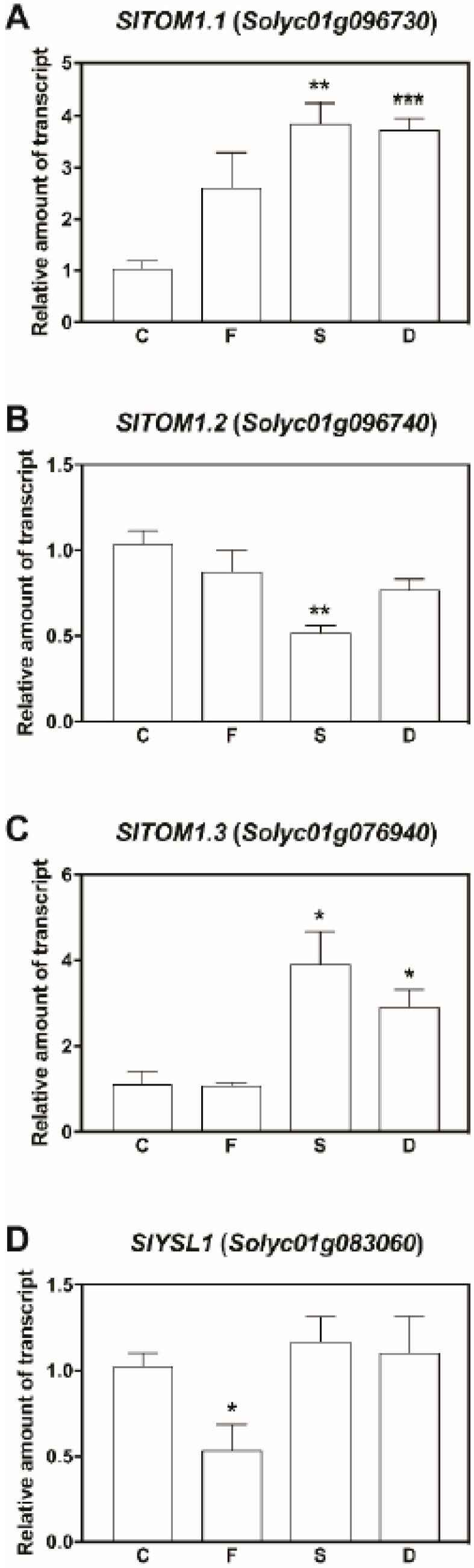
Gene expression analyses in the root tissue of tomato plants subjected to different nutritional regimes. The levels of expression of *SlTOM1.1* (**A**), *SlTOM1.2* (**B**), *SlTOM1.3* (**C**) and *SlYSL1* (**D**) were assessed by quantitative Real Time RT–PCR. The data were normalised to two internal controls: Tomato *LeEF-1* mRNA for elongation factor 1 alpha and Tomato *ubi3* gene for ubiquitin. The relative expression ratios were calculated using C plants as a calibrator sample. The values reported are means ± SE (*n* = 3). The statistical significance was tested by means Student′s t test (*, *p* < 0.05; **, *p* < 0.01; ***, *p* < 0.001).

The Transporter of Mugineic acid (TOM1) is the protein that mediates the release PS, belonging to the class of mugineic acid [31], which plays a pivotal role in Fe nutrition for Strategy II plants [31]. In fact, PSs can chelate Fe(III) in the rhizosphere, and afterwards the complexes Fe(III)-PSs are taken up through transporters belonging to the Yellow-Stripe-Like (YSL) gene families. The identification of hydroxymugineic acid in the exudome (Figure 6) prompted us to investigate whether tomato plants might feature molecular mechanisms involved in the exploitation of PSs for Fe nutrition. To this aim, genesputatively encoding orthologous *TOM1* transporters in *Solanum lycopersicum* genome were identified on the base of amino acid sequence similarity with members of the TOM1 transporters family in other organisms, like *Hordeum vulgare* and *Oryza sativa*, by running a Blastp analysis [38] in the *Solanum lycopersicum iTAG2.4* genome provided by Phytozome (http://phytozome.jgi.doe.gov/pz/portal.html). This approach allowed the retrieval of four transcripts, namely *Solyc01g096730.2.1*, *Solyc01g096720.2.1*, *Solyc01g096740.2.1* and *Solyc10g076940.1.1* (Figure S1). The phylogenetic analysis and a pairwise alignment highlighted that the sequences *Solyc01g096730.2.1* and *Solyc01g096720.2.1* identified the same transcript, therefore only *Solyc01g096730.2.1* was considered for the further molecular analyses (Figure S1). In order to understand whether the transcripts putatively encoding *SlTOM1* orthologous genes, *Solyc01g096730.2.1*, *Solyc01g096740.2.1* and *Solyc10g076940.1.1* (hereafter referred to as *SlTOM1.1, SlTOM1.2* and *SlTOM1.3*, respectively), might be involved in the plant responses to the different nutritional regimes, quantitative real time RT-PCR analyses were run. The expression of both *SlTOM1.1* and *SlTOM1.3* was significantly upregulated by about three to four times in S and D nutritional regimes (Figure 7A,C). On the contrary, the expression of *SlTOM1.2* was strongly reduced by the lack of sulfur in the growth medium (S condition), whilst the double deficiency did not modulate the gene transcription (Figure 7B). Noteworthy, the Fe deficiency condition did not induce the transcriptional regulation of any of the three *SlTOM1* genes (Figure 7).

The Yellow-Stripe-Like (YSL) protein sequences of *A. thaliana*, *O. sativa*, *Z. mays* and *Arachis hypogaea* have been used to isolate putative *YSL* orthologous transcript in the tomato *iTAG2.4* genome by running a Blastp analysis [38]. The analysis allowed the isolation of nine transcripts, namely *Solyc08g083060.2.1*, *Solyc09g074960.1.1*, *Solyc03g031920.2.1*, *Solyc02g005340.2.1*, *Solyc05g053110.1.1*, *Solyc03g082620.2.1*, *Solyc05g053110.1.1*, *Solyc02g094280.2.1* and *Solyc02g081570.2.1*, which have been subjected to multiple alignment and phylogenetic analysis. The phylogenetic tree displayed that one member (i.e., *Solyc08g083060.2.1,* hereafter referred to as *SlYSL1*) of this multigenic family of tomato had a high similarity with *AhYSL1* (Figure S2), which has already been characterized for its expression in the root tissues (epidermis/exodermis) of Fe-deficient peanut plants [39]. In addition, *AhYSL1* was also hypothesized to transport the Fe-DMA complexes inside peanut roots [39]. The quantitative real time RT-PCR analyses showed that *SlYSL1* expression was not modulated in S and D conditions in comparison with control roots (Figure 7D). On the other hand, a significant downregulation (about 50% reduction) was observed in plants grown in a Fe-deficient nutritional regime (F) when compared to control plants (Figure 7D).

## 3. Discussion

The root acquisition of both iron (Fe) and sulfur (S) has been extensively studied in crops, with these two nutrients being crucial for the vegetative development of plants and fundamental for the qualitative and quantitative features of their edible parts [40]. Considering the progressive depletion of S in the arable soil, ascribable to the reduction in S-rich industrial emissions [17,41], this topic is attracting very high attention. Interestingly, studies in the literature have also provided evidence that Fe and S nutrition are tightly linked; in fact, S is required for the biosynthesis, among other compounds, of the amino acid methionine, which is the precursor of both nicotianamine (NA) and mugineic acid (MA) [42]. Indeed, this latter, together with its derivatives (i.e., PS), is a key element in the Fe acquisition mechanism in Strategy II plants [31], whereas NA, along with citric acid, is required in both monocots and dicots for the chelation and the translocation of Fe within plant tissues [43]. Furthermore, S shortage has been also shown to limit the onset of Fe-deficiency response in dicot plants such as tomato [44]. Consistently, it has been demonstrated that a super-optimal S provision can help recover Fe deficiency symptoms in wheat, i.e., a monocot plant species, via an improved Fe use efficiency within the plant [45].

The results presented here show that imposition of Fe shortage significantly reduced the levels of chlorophyll content (Figure 2), whereas single S deficiency both in single and combined (S and Fe) condition (S and D condition, respectively) increased root growth (Figure 1). Leaf chlorosis is a typical phenomenon associated with Fe deficiency [40], whereas the increased development of root apparatus reflects a classical response to S limitation, as extensively reported in the literature [46]. Moreover, Fe deficiency (F condition) also limited the biomass accumulation at the shoot level (Figure 1), as previously described [44].

Despite previous studies widely demonstrating that root exudation is a fundamental tool adopted by plants to directly shape the rhizosphere favoring the root Fe acquisition [34,47,48], the knowledge about the root exudation pattern of S-deficient plants is rather limited. Furthermore, although the relationship between Fe and S uptake has been clearly demonstrated [11,12,13,14,15], to the best of our knowledge information concerning the exudation profiles of plants exposed to single S deficiency or combined Fe and S deficiency is still missing. Interestingly, Astolfi et al. [49] demonstrated that S starvation strongly limits the release of PSs in Fe-deficient barley plants, suggesting that the intracellular S levels most likely have an impact on the qualitative and quantitative root exudation pattern.

An untargeted metabolomic approach was undertaken using the complementary GC-MS and UHPLC/QTOF-MS techniques combined with multivariate statistical analysis methods, to acquire and analyze the exudomic profile of tomato plants subjected to Fe and S deficiency, alone or in combination. Interestingly, the PLS-DA score plots described four independent clusters, in accordance with the different nutritional regimes: control (C), single Fe (F) and S deficiency (S) and combined Fe and S deficiency (D) (Figure 3). However, the exudate profile of S condition was similar to that of control (Figure 3). These results strengthened the finding that both single Fe and S, and combined Fe-S starvations, induced a clearly distinct response, as previously reported at transcriptomic [50,51] and metabolomic [12] levels. Venn diagrams following Volcano analysis (*p*< 0.001, fold-change cut-off = 3) showed that the majority of the differentially represented metabolites were shared between the nutritional condition imposed (100 metabolites) (Figure 4). Furthermore, it revealed that 23% (51), 10% (21) and 21% (45) of differential metabolites were distinctively and significantly modulated by single Fe, single S and combined Fe-S deficiency, respectively (Figure 4). This study clearly confirms that plant roots secrete a wide range of compounds when exposed to a nutritional stress, improving the edaphic conditions (e.g., nutrient availability) in the rhizosphere. However, the types and the concentrations of the compounds released by the roots are considerably different between the four treatments. In this respect, it is intriguing to note that single Fe deficiency had a more pronounced impact on exudomic profile than that induced by S or the combined Fe-S deficiency.

We also performed the heatmaps containing the hierarchical cluster and the Pearson′s correlations supporting the results explained in the manuscript. Both the figures are provided as Supplementary Figure S4.

Due to the large dataset provided by the metabolomic study, an approach of data reduction based on the clustering by classes has been used in this work. Therefore, the encoded chemical structures were used to clusterized and define the different metabolites. Since some compounds are not yet classified into chemical ontologies, chemical similarity enrichment has been selected to cluster the principal classes of metabolites. In this regard, Tanimoto substructure chemical similarity coefficients were used to cluster metabolites into non-overlapping chemical groups in order to better understand the complexity of the metabolite signatures. Statistical significance was evaluated by the Kolmogorov–Smirnov test [37]. ChemRICH set enrichment statistics plot identified different chemical clusters, being phenols, coumaric acids and amino acids the most represented chemical clusters in tomato root exudates. This pattern was independent from the growing condition, albeit showing different variations according to the starvations (Figure 5). In particular, phenols and coumaric acids showed a general increase in all the conditions; indeed, the exudation of phenols has already been shown to be involved in the response to Fe starvation, possibly for the mobilization of sparingly soluble Fe sources [52,53,54,55]. Moreover, phenolics can increase the solubility and the uptake of metals such as Fe by chelation or reduction [52,54,55,56]. Similar results were found by other authors who showed that the *Arabidopsis* phenylpropanoid pathway is stimulated in roots by Fe-deficiency [54,57]. oumarins have already been shown to be part of the Fe deficiency response in plants. In particular, the biosynthesis and the release of catechol-type coumarins have been demonstrated to contribute to Fe acquisition under limiting conditions [58,59,60,61,62,63,64,65,66]. Interestingly, both phenolic compounds and coumarins were also released in S and D conditions, possibly highlighting that this mechanism of the Fe deficiency response is not affected by the inhibitory effect induced by S shortage on the Fe nutrient acquisition process.

On the contrary, the class of amino acids increased only in the root exudates of F plants. At present, very little is known about the role of these compounds in root exudates as a response to nutrient starvation; to date, the release of amino acids has been observed in P-deficient soybean and strawberry plants [34,67] and in cucumber plants subjected to Fe deficiency [33]. Among the overrepresented amino acids, glycine and glutamic acid were the only two compounds specifically over-accumulated in the root exudates of F plants, as also previously reported by [33]. These pieces of evidence further strengthen the hypothesis that glycine might participate in the Fe mobilization from barely available sources via a complexation process. In fact, the stability constant of the complex Fe(III)-glycine is very similar to that of Fe(III)-citrate [33]. On the other hand, glutamic acid has been described as a strong chemoattractant for bacteria at rhizosphere level [48,68]; therefore, its presence in the root exudates might suggest that Fe-starved tomato plants are actively recruiting the rhizosphere microbiota in favor of useful associations, i.e., with plant growth-promoting rhizobacteria, to cope more efficiently with the nutrient disorder [69].

A particularly intriguing aspect of this study is represented by the results obtained analysing the root exudation profiles of tomato plants grown in conditions of adequate availability of Fe and S (C condition). In fact, in this case, the presence of 3-hydroxymugineic acid, an organic compound belonging to the class of PSs, has been detected. Moreover, its amount is considerably decreased (4-fold decrease) when the plants have been exposed only to the single Fe or single S deficiency. No changes have been measured in its levels in exudates of dual (Fe-S) deficient plants, compared to control (Figure 6). Concerning this specific result, it is well known that Fe-starved plants secrete in the rhizosphere a diversity of compounds playing an important role in the biogeochemical cycle of this element. In fact, thanks to the Fe mobilization process, the extent of the nutrient solubilization from soil minerals and the magnitude of its uptake by roots can be significantly increased. In particular, it has been shown that Strategy I plants release phenolics from roots under Fe deficiency [34,56,70], whereas the secretion of PS is typical of Strategy II plants (only grasses). Our finding strongly supports the hypothesis that the distinction between the two mechanisms/strategies could not be so sharp [71]. The results of this study emphasize that the two strategies are not mutually exclusive and report that a MA derivative was actually detected in root exudates of dicot plants. Therefore, such information suggests the existence, also in tomato, of a double strategy for Fe acquisition, as already demonstrated in rice plants [31]. Indeed, recent pieces of research showed that Fe deficiency symptoms in *Arachis hypogea* plants could be alleviated by the intercropping with maize plants [39]. In particular, these authors demonstrated that the deoxymugineic acid (DMA) released by maize plants could bind Fe(III) in the soil and the complex DMA-Fe(III) could be taken up by peanut plants through AhYSL1 transporters [39]. In Strategy II plants, the release of PSs is mediated by transporters *TOM1* exposed on the plasma membrane of the root cells that has been extensively characterized in graminaceous plants [31]. Interestingly, the phylogenetic analyses tomato genome highlighted the presence of at least three *TOM1* gene putative isoforms (*SlTOM1.1*, *SlTOM1.2* and *SlTOM1.3*) for the release of PSs and one *SlYSL1* gene homologous to *AhYSL1*, putatively involved in the uptake of the Fe(III)-PSs complexes. The gene expression analyses showed that *SlTOM1.2* was the most expressed gene among the *TOM1* homologs (Figure S3) and its expression was highest in controls. On the contrary, the expression of this gene was significantly downregulated in single Fe, single S and combined Fe-S deficiency, supporting the decrease in 3-hydroxymugineic acid contents measured at the metabolomic level (Figure 6 and Figure 7). Notably, *SlYSL1* was significantly down-regulated in F plants (Figure 7d).

These data might suggest that in tomato plants both strategy I and II are encoded at the genomic level and expressed concurrently only when the availability of the two nutrients (i.e., Fe and S) is not limited. On the contrary, when Fe is lacking, tomato plants were shown to rely just on one mechanism (Strategy I), probably the most efficient way to acquire the micronutrient from the external environment. As a consequence, the molecular entities involved in the Strategy II are then shut down (i.e., synthesis of 3-hydroxymugineic acid and restraint of the *SlTOM1.2* and *SlYSL1* gene expression).

**Figure 8:**
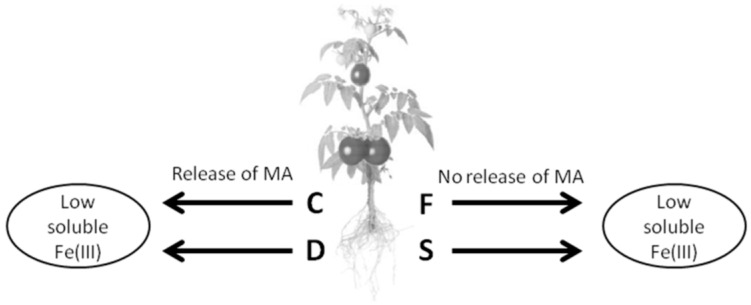
Scheme showing suggested Fe-solubilizing mechanisms occurring in rhizosphere when tomato plants were grown in complete nutrient solution (control, C) or under Fe (F), sulfur (S) and combined Fe + S deficiency (D).

In conclusion, the evidence here reported seems to support the hypothesis of a co-existence of the two Fe acquisition strategies in tomato plants (Figure 8). However, these data do not rule out a specificity of the response to the nutritional disorder in tomato. In fact, while the exposure of tomato plants to the single Fe deficiency is associated to a decreased secretion of 3-hydroxymugineic acid (Figure 6 and Figure 8), this compound is considerably accumulated in Strategy II plants as a consequence of the same nutritional stress [72]. It can be postulated that a strategy shift, towards the more efficient one for coping with the Fe shortage (i.e., Strategy I in tomato plants), could be at the basis of this behavior. Moreover, the strongly reduced secretion of hydroxymugineic acid by tomato roots under S-deficient conditions confirms the crucial role played by the intracellular S pools in its synthesis [49] and, more generally, in the biogeochemical cycle of Fe in the rhizosphere.

## 4. Materials and Methods

### 4.1. Experimental Conditions and Treatments

The experiment was conducted with hydroponically grown tomato (*Solanum lycopersicum* L. cv. Marmande) plants. Seeds were germinated for six days in darkness at 28 °C and, after germination, uniform seedlings were transferred to plastic pots (six seedlings per pot) containing 2.2 L of nutrient solution [73] for seven days, being exposed to 1.2 mM sulfate and 40 µM Fe^III^-EDTA. Plants were then transferred for a further nine days to four different nutritional conditions (control, C, 1.2 mM sulfate and 40 μM FeIII-EDTA; Fe deficiency, F, 1.2 mM sulfate and 0 μM FeIII-EDTA; S deficiency, S, 0 mM sulfate and 40 μM FeIII-EDTA; dual deficiency, D, 0 mM sulfate and 0 μM FeIII-EDTA).

In S-free nutrient solution, sulphate salts (K^+^, Mn^2+^, Zn^2+^, Cu^2+^) were replaced by appropriate amounts of the corresponding chloride salts (K^+^, Mn^2+^, Zn^2+^, Cu^2+^). Nutrient solution was continuously aerated and changed every 3 days. Plants were grown in a growth chamber under 200 µmol photons m^−2^ s^−1^ PPF and 14 h/10 h day/night regime (27/20 °C day/night temperature cycling; 80% relative humidity). Both leaves and roots were harvested 16 days after transfer in nutrient solution.

### 4.2. Collection of Root Exudates

Root exudates were collected nine days after the imposition of various treatments (C, F, S and D). Briefly, tomato plants were removed from the nutrient solution and the roots were carefully washed for 30 min in 2 L of deionised water. Then, the root systems of four plants from each condition (about 0.5 g root fresh weight) were immersed into 10 mL deionised water for 6 h under continuous aeration. Thereafter, Micropur (10 mg L^−1^) (Roth, Karlsruhe, Germany) was added to prevent microbial degradation of organic compounds.

### 4.3. Metabolomic Profiling by UHPLC/QTOF-MS

Root exudates were filtered through a 0.22 μm cellulose membrane into an amber vial for analysis. The profile of metabolites was determined through UHPLC liquid chromatography with quadrupole-time-of-flight mass spectrometry (UHPLC/QTOF-MS). In more detail, a 1290 series LC system equipped with a binary pump, an JetStream Electrospray source and a G6550 iFunnel QTOF mass spectrometer (Agilent technologies, Santa Clara, CA, USA) were used as previously reported [74]. Briefly, the QTOF acquisition was carried out in positive SCAN mode (100–1200 m/z range) and a chromatographic gradient reverse phase separation was achieved using water and methanol as eluents on an Agilent Zorbax Eclipse-plus column (75 × 2.1 mm i.d., 1.8 μm). The injection volume was 3.5 μL and flow rate was 220 μL min^−1^ [75]. Feature deconvolution as well as the following mass and retention time alignments were carried out in Profinder B.06 (from Agilent Technologies). Compound annotation was based on accurate mass (mass accuracy < 5 ppm), isotope spacing and isotope ratio, against a custom database produced by combining compounds exported from PlantCyc 9.6 (Plant Metabolic Network, http://www.plantcyc.org; accessed April 2017), Phenol-Explorer 3.6 (http://www.phenol-explorer.eu; accessed April 2017), as well as additional compounds taken from the literature and that might be present in root exudates. Based on the approach used, the annotation level corresponded to Level 2 (putatively annotated compounds) of COSMOS Metabolomics Standards Initiative (http://cosmos-fp7.eu/msi.html).

### 4.4. Metabolomic Profiling by GC/MS

A further gas chromatography mass spectrometry (GC/MS) analysis [76] was also applied to achieve complementary information about chemical profiles in root exudates. Briefly, an aliquot (200 µL) of root exudate was dried overnight in a speed vacuum concentrator. Thereafter, 60 µL of 2% methoxyamine hydrochloride (Sigma-Aldrich, St Louis, MO, USA) in pyridine, 90 µL of N-Methyl-N-(trimethylsilyl) trifluoroacetamide and 1% trimethyl chlorosilane (Sigma-Aldrich) were added for derivatization purposes [77]. Finally, the samples were transferred into vials to be analysed.

An Agilent 6890 gas chromatograph coupled to an Agilent 5977 quadrupole mass spectrometer were used for GC/MS analysis. Chromatographic separation was done on a 30 m DB-5MS capillary column. Derivatized extracts were injected in splitless mode (250 °C), and helium (1 mL/min) was used as carrier gas. The oven temperature program started from 100 °C (maintained for 2 min) up to 325 °C at 10 °C min^−1^. A fatty acid methyl ester mixture (FAME mix, Agilent Technologies) was used to calculate retention indexes. Deconvolution was done using the software ‘Unknown Analysis′ (Agilent Technologies) and identification was based on spectral comparison against the ‘Fiehn Library′ (https://fiehnlab.ucdavis.edu/projects/fiehnlib, Agilent Technologies, released May 2016).

### 4.5. Bioinformatics Analysis

The *TOM* sequences of *Hordeum vulgare* (*HvTOM1*) and *Oryza sativa* (*OsTOM1*, *OsTOM2* and *OsTOM3*) were used to obtain *SlTOM* homologues by an amino acids sequence BLAST analysis, using the *Solanum lycopersicum iTAG2.4* genome provided by Phytozome (http://phytozome.jgi.doe.gov/pz/portal.html). Similarly, the Yellow Stripe-like (YSL) protein sequences from *Hordeum vulgare*, Zea mays, *Oryza sativa and Arachis hypogea* were used to obtain *SlYSL* homologues by an amino acids sequence BLAST analysis, using the *Solanum lycopersicum iTAG2.4* genome. Subsequently, multiple sequence alignments were run by CLUSTALW algorithm (http://clustalw.ddbj.nig.ac.jp/) and the phylogenetic trees were obtained using FigTree (http://taxonomy.zoology.gla.ac.uk/rod/treeview.html) for both TOM and YSL transporters.

### 4.6. Extraction of Total RNA, cDNA Preparation and Expression Analyses

Total RNA was extracted from frozen tomato root tissues using the Spectrum Plant Total RNA Kit (Sigma Aldirch). Then, 1 µg of RNA was treated with 1ug of DNAse RQ1 (Promega, Madison, WI, USA). The cDNA synthesis was performed using ImProm-II Reverse Transcription System (Promega, Madison, WI, USA). Quantitative Real-Time RT-PCR was performed using gene-specific primers (approximately 20 nt-long), amplifying fragments of about 100 bp (Table S1). The specificity of each primer was tested, firstly performing a BLAST directly on the tomato genome mRNA prediction and subsequently carrying out the melting curve. The elongation factor and the ubiquitin primers were obtained by Vigani et al. [14]. The Real-Time RT PCR was performed using SsoFast EvaGreen Supermix (Bio-Rad, Hercules, California 94547, USA), the Qiagen Rotor Gene Q Real-Time PCR. The amplification efficiency was obtained through LinReg PCR [78] and the relative expression and the standard error was calculated according to Paffl, Horgan and Dempfle [79].

### 4.7. Other Measurements

The measurements of chlorophyll levels per unit area were estimated in attached leaves with a non-destructive portable apparatus, the SPAD chlorophyll meter (Minolta Co.), using the youngest fully expanded leaf from the top of each plant and presented as SPAD units.

### 4.8. Statistical Analysis

Each reported value represents the mean ± SD of measurements carried out in triplicate and obtained from four independent experiments. Statistical analyses of data were carried out by ANOVA with the GraphPad InStat Program (version 3.06). Significant differences were established by posthoc comparisons (HSD test of Tukey) at *p* < 0.01.

Both GC/MS and UHPLC/QTOF-MS metabolomics data were interpreted in Agilent Mass Profiler Professional B.12.06 (from Agilent Technologies, https://www.agilent.com/en/products/software-informatics/mass-spectrometry-software/data-analysis/mass-profiler-professional-software). Post-acquisition processing included normalization at the 75th percentile and baselining to the median of the control. Unsupervised hierarchical cluster analysis (Euclidean similarity, ‘Wards′ linkage rule) was formerly carried out on the basis heatmaps generated from fold-change values. Thereafter, Partial Least Squares Discriminant Analysis (PLS-DA) supervised modelling was also done. The metabolites having the highest discrimination potential, based on model prediction scores on first and second latent vectors, were identified. Analysis of variance (one-way ANOVA, Benjamini-Hochberg multiple testing correction) and fold-change analysis (cut-off: 3-fold) were finally done in Mass Profiler Professional.

Chemical Similarity Enrichment Analysis (ChemRICH) was performed, as previously described [37]. The online web-app tool (http://chemrich.fiehnlab.ucdavis.edu) was used for this purpose. Therein, Tanimoto substructure chemical similarity coefficients were used to cluster metabolites into non-overlapping chemical groups. 

Metabolomic data were also analysed using the software Metaboanalyst 4.0 (https://www.metaboanalyst.ca/) for hierarchical clustering and correlation analysis. Data, expressed as peak intensity, were transformed through “Log normalization” and scaled through auto-scaling. A heat map was performed by hierarchical clustering by adopting the Ward′s clustering algorithm and the Euclidean distance and correlation analysis using the Pearson′s r. 

Multivariate Analysis of Variance (MANOVA) among the treatments was performed using PAST statistic software version 4.02 [80].

## Supplementary Materials

Supplementary materials can be found at https://www.mdpi.com/1422-0067/21/11/4038/s1.

## References

[1] Scherr S.J. Soil Degradation: A Threat to Developing Country Food Security by 2020?. https://www.researchgate.net/profile/Sara_Scherr/publication/5055892_Soil_Degradation_A_Threat_to_Developing-Country_Food_Security_by_2020/links/556307bf08ae6f4dcc954b8e/Soil-Degradation-A-Threat-to-Developing-Country-Food-Security-by-2020.pdf.

[2] White P.J., Brown P.H. (2010). Plant nutrition for sustainable development and global health. Ann. Bot..

[3] Tilman D., Cassman K.G., Matson P., Naylor R., Polasky S. (2002). Agricultural sustainability and intensive production practices. Nature.

[4] Taiz L. (2013). Agriculture, plant physiology, and human population growth: Past, present, and future. Theor. Exp. Plant. Physiol..

[5] Goucher L., Bruce R., Cameron D.D., Lenny Koh S.C., Horton P. (2017). The environmental impact of fertilizer embodied in a wheat-to-bread supply chain. Nat. Plants.

[6] Sambo P., Nicoletto C., Giro A., Pii Y., Valentinuzzi F., Mimmo T., Lugli P., Orzes G., Mazzetto F., Astolfi S. (2019). Hydroponic Solutions for Soilless Production Systems: Issues and Opportunities in a Smart Agriculture Perspective. Front. Plant Sci..

[7] Tomasi N., Mimmo T., Terzano R., Alfeld M., Janssens K., Zanin L., Pinton R., Varanini Z., Cesco S. (2014). Nutrient accumulation in leaves of Fe-deficient cucumber plants treated with natural Fe complexes. Biol. Fertil. Soils.

[8] Pii Y., Cesco S., Mimmo T. (2014). Shoot ionome to predict the synergism and antagonism between nutrients as affected by substrate and physiological status. Plant Physiol. Biochem..

[9] Marastoni L., Sandri M., Pii Y., Valentinuzzi F., Brunetto G., Cesco S., Mimmo T. (2019). Synergism and antagonisms between nutrients induced by copper toxicity in grapevine rootstocks: Monocropping vs. intercropping. Chemosphere.

[10] Marastoni L., Sandri M., Pii Y., Valentinuzzi F., Cesco S., Mimmo T. (2019). Morphological Root Responses and Molecular Regulation of Cation Transporters Are Differently Affected by Copper Toxicity and Cropping System Depending on the Grapevine Rootstock Genotype. Front. Plant Sci..

[11] Forieri I., Wirtz M., Hell R. (2013). Towards new perspectives on the interaction of iron and sulfur metabolism in plants. Front. Plant Sci..

[12] Zuchi S., Watanabe M., Hubberten H.M., Bromke M., Osorio S., Fernie A.R., Celletti S., Paolacci A.R., Catarcione G., Ciaffi M. (2015). The interplay between sulfur and iron nutrition in tomato. Plant Physiol..

[13] Zamboni A., Celletti S., Zenoni S., Astolfi S., Varanini Z. (2017). Root physiological and transcriptional response to single and combined S and Fe deficiency in durum wheat. Env. Exp. Bot..

[14] Vigani G., Pii Y., Celletti S., Maver M., Mimmo T., Cesco S., Astolfi S. (2018). Mitochondria dysfunctions under Fe and S deficiency: Is citric acid involved in the regulation of adaptive responses?. Plant. Physiol. Biochem..

[15] Coppa E., Celletti S., Pii Y., Mimmo T., Cesco S., Astolfi S. (2018). Revisiting Fe/S interplay in tomato: A split-root approach to study the systemic and local responses. Plant Sci..

[16] Astolfi S., Pii Y., Terzano R., Mimmo T., Celletti S., Allegretta I., Lafiandra D., Cesco S. (2018). Does Fe accumulation in durum wheat seeds benefit from improved whole-plant sulfur nutrition?. J. Cereal Sci..

[17] Schnug E., Evans E.J. (1992). Monitoring of the sulfur supply of agricultural crops in northern Europe. Phyton.

[18] Colombo C., Palumbo G., He J.Z., Pinton R., Cesco S. (2014). Review on iron availability in soil: Interaction of Fe minerals, plants, and microbes. J Soils Sediments.

[19] Mimmo T., Del Buono D., Terzano R., Tomasi N., Vigani G., Crecchio C., Pinton R., Zocchi G., Cesco S. (2014). Rhizospheric organic compounds in the soil-microorganism-plant system: Their role in iron availability. Eur. J. Soil. Sci..

[20] Dakora F.D., Phillips D.A. (2002). Root exudates as mediators of mineral acquisition in low-nutrient environments. Plant Soil.

[21] Walker T.S., Bais H.P., Grotewold E., Vivanco J.M. (2003). Root exudation and rhizosphere biology. Plant Physiol..

[22] Badri D.V., Vivanco J.M. (2009). Regulation and function of root exudates. Plant Cell Environ..

[23] Canarini A., Kaiser C., Merchant A., Richter A., Wanek W. (2019). Root exudation of primary metabolites: Mechanisms and their roles in plant responses to environmental stimuli. Front. Plant Sci..

[24] Rengel Z., Marschner P. (2005). Nutrient availability and management in the rhizosphere: Exploiting genotypic differences. New Phytol..

[25] Houmani H., Rabhi M., Abdelly C., Debez A., Hakeem K. (2015). Implication of rhizosphere acidification in nutrient uptake by plants: Cases of potassium (K), phosphorus (P), and iron (Fe). Crop Production and Global Environmental Issues.

[26] Terzano R., Cesco S., Mimmo T. (2015). Dynamics, thermodynamics and kinetics of exudates: Crucial issues in understanding rhizosphere processes. Plant Soil.

[27] Terzano R., Cuccovillo G., Gattullo C.E., Medici L., Tomasi N., Pinton R., Mimmo T., Cesco S. (2015). Combined effect of organic acids and flavonoids on the mobilization of major and trace elements from soil. Biol. Fert. Soils.

[28] Gattullo C.E., Pii Y., Allegretta I., Medici L., Cesco S., Mimmo T., Terzano R. (2018). Iron mobilization and mineralogical alterations induced by iron-deficient cucumber (*Cucumis sativus* L.) plants in a calcareous soil. Pedosphere.

[29] Pii Y., Mimmo T., Tomasi N., Terzano R., Cesco S., Crecchio C. (2015). Microbial interactions in the rhizosphere: Beneficial influences of plant growth-promoting rhizobacteria on nutrient acquisition process. A review. Biol. Fert. Soils.

[30] Alegria Terrazas R., Giles C., Paterson E., Robertson-Albertyn S., Cesco S., Mimmo T., Pii Y., Bulgarelli D. (2016). Plant-microbiota interactions as a driver of the mineral turnover in the rhizosphere. Adv. App. Microbiol..

[31] Kobayashi T., Nishizawa N.K. (2012). Iron uptake, translocation, and regulation in higher plants. Annu. Rev. Plant Biol..

[32] Bocchini M., Bartucca M.L., Ciancaleoni S., Mimmo T., Cesco S., Pii Y., Albertini E., Del Buono D. (2015). Iron deficiency in barley plants: Phytosiderophore release, iron translocation, and DNA methylation. Front. Plant Sci..

[33] Pii Y., Penn A., Terzano R., Crecchio C., Mimmo T., Cesco S. (2015). Plant-microorganism-soil interactions influence the Fe availability in the rhizosphere of cucumber plants. Plant Physiol. Biochem..

[34] Valentinuzzi F., Pii Y., Vigani G., Lehmann M., Cesco S., Mimmo T. (2015). Phosphorus and iron deficiencies induce a metabolic reprogramming and affect the exudation traits of the woody plant *Fragaria × ananassa*. J. Exp. Bot..

[35] Celletti S., Pii Y., Mimmo T., Cesco S., Astolfi S. (2016). The characterization of the adaptive responses of durum wheat to different Fe availability highlights an optimum Fe requirement threshold. Plant Physiol. Biochem..

[36] Valentinuzzi F., Cesco S., Tomasi N., Mimmo T. (2015). Influence of different trap solutions on the determination of root exudates in *Lupinus albus* L.. Biol. Fert. Soils.

[37] Barupal D.K., Fiehn O. (2017). Chemical Similarity Enrichment Analysis (ChemRICH) as alternative to biochemical pathway mapping for metabolomic datasets. Sci. Rep..

[38] Altschul S.F., Madden T.L., Schäffer A.A., Zhang J., Zhang Z., Miller W., Lipman D.J. (1997). Gapped BLAST and PSI-BLAST: A new generation of protein database search programs. Nucleic Acids Res..

[39] Xiong H., Kakei Y., Kobayashi T., Guo X., Nakazono M., Takahashi H., Nakanishi H., Shen H., Zhang F., Nishizawa N.K. (2013). Molecular evidence for phytosiderophore-induced improvement of iron nutrition of peanut intercropped with maize in calcareous soil. Plant Cell Environ..

[40] Marschner H., Marschner P. (2012). Marschner′s Mineral Nutrition of Higher Plants.

[41] Scherer H.W. (2001). Sulphur in crop production—invited paper. Eur. J. Agron..

[42] Hesse H., Hoefgen R. (2003). Molecular aspects of methionine biosynthesis. Trends Plant Sci..

[43] Kobayashi T., Nozoye T., Nishizawa N.K. (2019). Iron transport and its regulation in plants. Free Radic. Biol. Med..

[44] Zuchi S., Cesco S., Varanini Z., Pinton R., Astolfi S. (2009). Sulphur deprivation limits Fe deficiency responses in tomato plants. Planta.

[45] Celletti S., Paolacci A.R., Mimmo T., Pii Y., Cesco S., Ciaffi M., Astolfi S. (2016). The effect of excess sulfate supply on iron accumulation in three graminaceous plants at the early vegetative phase. Environ. Exp. Bot..

[46] Nikiforova V.J., Gakière B., Kempa S., Adamik M., Willmitzer L., Hesse H., Hoefgen R. (2004). Towards dissecting nutrient metabolism in plants: A systems biology case study on sulphur metabolism. J. Exp. Bot..

[47] Rellán-Álvarez R., Andaluz S.S., Rodríguez-Celma J., Wohlgemuth G., Zocchi G., Álvarez-Fernández A., Fiehn O., López-Millán A.F., Abadía J. (2010). Changes in the proteomic and metabolic profiles of *Beta vulgaris* root tips in response to iron deficiency and resupply. BMC Plant Biol..

[48] Carvalhais L.C., Dennis P.G., Fedoseyenko D., Hajirezaei M.R., Borriss R., von Wirén N. (2011). Root exudation of sugars, amino acids, and organic acids by maize as affected by nitrogen, phosphorus, potassium, and iron deficiency. J. Plant Nut. Soil Sci..

[49] Astolfi S., Cesco S., Zuchi S., Neumann G., Roemheld V. (2006). Sulfur starvation reduces phytosiderophores release by iron-deficient barley plants. Soil Sci. Plant Nut..

[50] Watanabe M., Hubberten H.M., Saito K., Hoefgen R. (2010). General regulatory patterns of plant mineral nutrient depletion as revealed by serat quadruple mutants disturbed in cysteine synthesis. Mol. Plant.

[51] Watanabe M., Hubberten H.M., Hoefgen R., De Kok L.J., Tabe L., Tausz M., Hawkesford M.J., Hoefgen R., McManus M.T., Norton R., Rennenberg H., Saito K. (2012). Plant Response to Mineral Ion Availability: Transcriptome Responses to Sulfate, Selenium and Iron. Sulfur Metabolism in Plants: Mechanisms and Applications to Food Security and Responses to Climate Change.

[52] El-Baz F.K., Mohamed A.A., Aboul-Enein A.M., Salama Z.A. (2004). Alteration in root exudates level during Fe-deficiency in two cucumber cultivars. Int. J. Agric. Biol..

[53] Tomasi N., Weisskopf L., Renella G., Landi L., Pinton R., Varanini Z., Nannipieri P., Torrent J., Martinoia E., Cesco S. (2008). Flavonoids of white lupin roots participate in phosphorus mobilization from soil. Soil. Biol. Biochem..

[54] Chen Y.T., Wang Y., Yeh K.C. (2017). Role of root exudates in metal acquisition and tolerance. Curr. Opin. Plant Biol..

[55] Schmidt M.A., Halvorson J.J., Hagerman A.E., Gonzalez J.M. (2017). Macronutrients and metals released from soils by solutions of naturally occurring phenols. J. Plant Nutr. Soil Sci..

[56] Cesco S., Neumann G., Tomasi N., Pinton R., Weisskopf L. (2010). Release of plant-borne flavonoids into the rhizosphere and their role in plant nutrition. Plant Soil.

[57] Yang T.J.W., Lin W.D., Schmidt W. (2010). Transcriptional profiling of the Arabidopsis iron deficiency response reveals conserved transition metal homeostasis networks. Plant Physiol..

[58] Rodríguez-Celma J., Lin W.D., Fu G.M., Abadía J., López-Millán A.F., Schmidt W. (2013). Mutually exclusive alterations in secondary metabolism are critical for the uptake of insoluble iron compounds by *Arabidopsis* and *Medicago truncatula*. Plant Physiol..

[59] Fourcroy P., Sisó-Terraza P., Sudre D., Savirón M., Reyt G., Gaymard F., Abadía A., Abadia J., Álvarez-Fernández A., Briat J.F. (2014). Involvement of the ABCG37 transporter in secretion of scopoletin and derivatives by Arabidopsis roots in response to iron deficiency. New Phytol..

[60] Fourcroy P., Tissot N., Gaymard F., Briat J.F., Dubos C. (2016). Facilitated Fe Nutrition by Phenolic Compounds Excreted by the Arabidopsis ABCG37/PDR9 Transporter Requires the IRT1/FRO2 High-Affinity Root Fe^2+^ Transport System. Mol. Plant.

[61] Schmid N.B., Giehl R.F.H., Döll S., Mock H.P., Strehmel N., Scheel D., Kong X., Hider R.C., von Wirén N. (2014). Feruloyl-CoA 6′-Hydroxylase1-Dependent Coumarins Mediate Iron Acquisition from Alkaline Substrates in Arabidopsis. Plant Physiol..

[62] Schmidt H., Günther C., Weber M., Spörlein C., Loscher S., Böttcher C., Schobert R., Clemens S. (2014). Metabolome Analysis of Arabidopsis thaliana Roots Identifies a Key Metabolic Pathway for Iron Acquisition. PLoS ONE.

[63] Sisó-Terraza P., Luis-Villarroya A., Fourcroy P., Briat J.F., Abadía A., Gaymard F., Abadía J., Álvarez-Fernández A. (2016). Accumulation and secretion of coumarinolignans and other coumarins in *Arabidopsis thaliana* roots in response to iron deficiency at high pH. Front. Plant Sci..

[64] Siwinska J., Siatkowska K., Olry A., Grosjean J., Hehn A., Bourgaud F., Meharg A.A., Carey M., Lojkowska E., Ihnatowicz A. (2018). Scopoletin 8-hydroxylase, A novel enzyme involved in coumarin biosynthesis and iron-deficiency responses in Arabidopsis. J. Exp. Bot..

[65] Rajniak J., Giehl R.F.H., Chang E., Murgia I., von Wirén N., Sattely E.S. (2018). Biosynthesis of redox-active metabolites in response to iron deficiency in plants. Nat. Chem. Biol..

[66] Tsai H.H., Rodríguez-Celma J., Lan P., Wu Y.C., Vélez-Bermúdez I.C., Schmidt W. (2018). Scopoletin 8-Hydroxylase-Mediated Fraxetin Production Is Crucial for Iron Mobilization. Plant Physiol..

[67] Tawaraya K., Horie R., Saito S., Wagatsuma T., Saito K., Oikawa A. (2014). Metabolite Profiling of Root Exudates of Common Bean under Phosphorus Deficiency. Metabolites.

[68] Barbour W.M., Hattermann D.R., Stacey G. (1991). Chemotaxis of *Bradyrhizobium japonicum* to soybean exudates. Appl. Environ. Microbiol..

[69] Pii Y., Borruso L., Brusetti L., Crecchio C., Cesco S., Mimmo T. (2016). The interaction between iron nutrition, plant species and soil type shapes the rhizosphere microbiome. Plant Physiol. Biochem..

[70] Mimmo T., Ghizzi M., Marzadori C., Gessa C.E. (2008). Organic acid extraction from rhizosphere soil: Effect of field-moist, dried and frozen samples. Plant Soil.

[71] Grillet L., Schmidt W. (2019). Iron acquisition strategies in land plants: Not so different after all. New Phytol..

[72] Nozoye T., Nagasaka S., Kobayashi T., Takahashi M., Sato Y.Y., Uozumi N., Nakanishi H., Nishizawa N.K. (2011). Phytosiderophore efflux transporters are crucial for iron acquisition in graminaceous plants. J. Biol. Chem..

[73] Zhang F.S., Römheld V., Marschner H. (1991). Role of the root apoplasm for iron acquisition by wheat plants. Plant Physiol..

[74] Kumar P., Lucini L., Rouphael Y., Cardarelli M., Kalunke R.M., Colla G. (2015). Insight into the role of grafting and arbuscular mycorrhiza on cadmium stress tolerance in tomato. Front. Plant Sci..

[75] Lucini L., Borgognone D., Rouphael Y., Cardarelli M., Bernardi J., Colla G. (2016). Mild potassium chloride stress alters the mineral composition, hormone network, and phenolic profile in artichoke leaves. Front. Plant Sci..

[76] Lucini L., Colla G., Miras Moreno M.B., Bernardo L., Cardarelli M., Terzi V., Bonini P., Rouphael Y. (2019). Inoculation of *Rhizoglomus irregulare* or *Trichoderma atroviride* differentially modulates metabolite profiling of wheat root exudates. Phytochemistry.

[77] Lisec J., Schauer N., Kopka J., Willmitzer L., Fernie A.R. (2006). Gas chromatography mass spectrometry-based metabolite profiling in plants. Nat. Protoc..

[78] Ramakers C., Ruijter J.M., Deprez R.H.L., Moorman A.F.M. (2003). Assumption-free analysis of quantitative real-time polymerase chain reaction (PCR) data. Neurosci. Lett..

[79] Pfaffl M.W., Horgan G.W., Dempfle L. (2002). Relative expression software tool (REST©) for group-wise comparison and statistical analysis of relative expression results in real-time PCR. Nucleic. Acids. Res..

[80] Hammer Ø., Harper D.A.T., Ryan P.D. (2001). PAST: Paleontological statistics software package for education and data analysis. Palaeontol. Electron..

