# Supplementary material for: Single and Combined Fe and S Deficiency Differentially Modulate Root Exudate Composition in Tomato: A Double Strategy for Fe Acquisition?
Source: Int J Mol Sci. 2020 Jun 5;21(11):4038. doi: 10.3390/ijms21114038 (PMC7312093; doi:10.3390/ijms21114038)
Supplement: Supplementary file 1 [file ijms-21-04038-s001.zip › Supplementary Figure 4.docx]

**A B**

| 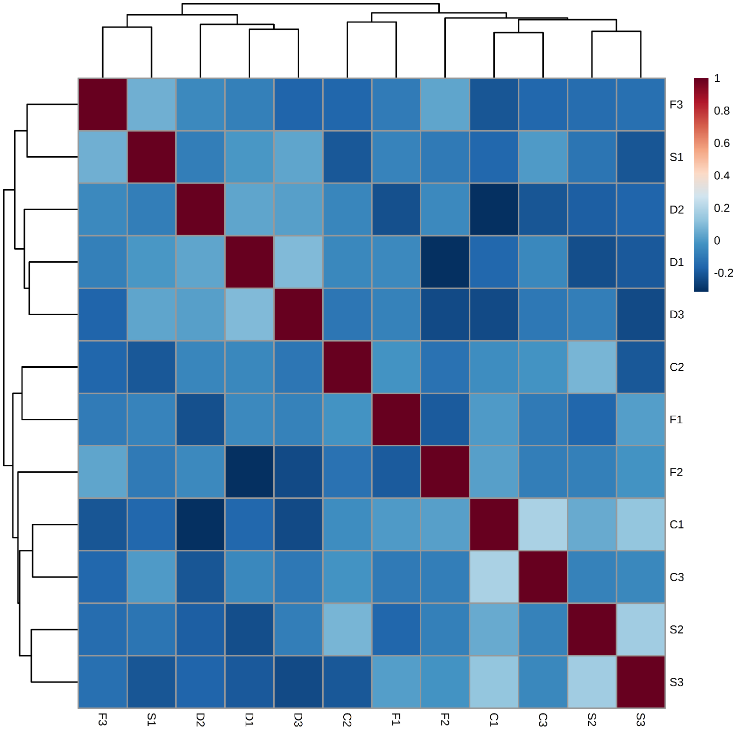 | 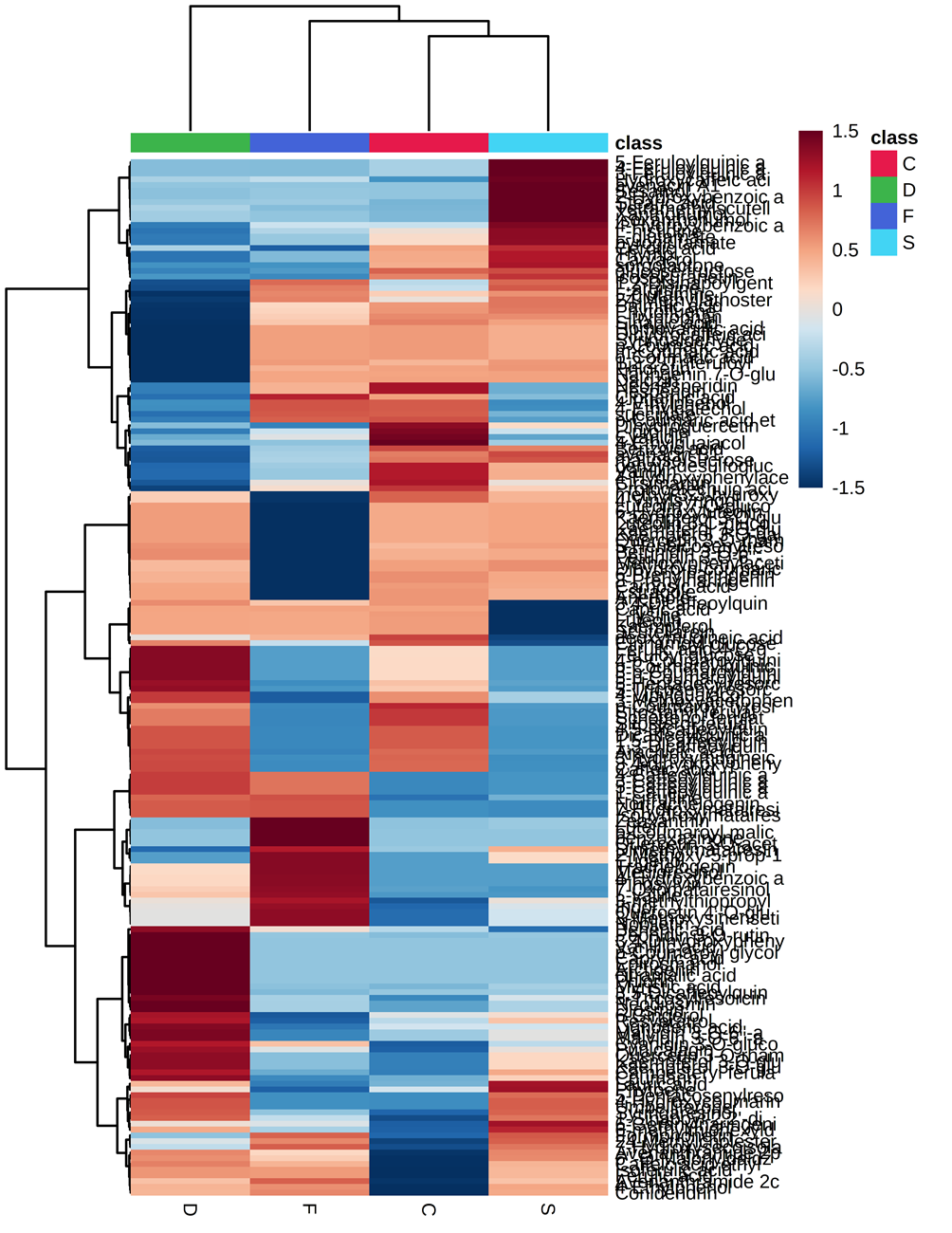 |
| --- | --- |

**Supplementary Figure 4.** Heatmaps containing a) the hierarchical cluster and b) the Pearson's correlations.
