# Supplementary material for: Single and Combined Fe and S Deficiency Differentially Modulate Root Exudate Composition in Tomato: A Double Strategy for Fe Acquisition?
Source: Int J Mol Sci. 2020 Jun 5;21(11):4038. doi: 10.3390/ijms21114038 (PMC7312093; doi:10.3390/ijms21114038)
Supplement: Supplementary file 1 [file ijms-21-04038-s001.zip › Supplementary Table 1.docx]

**Supplementary Table 1. Sequences of the gene specific primenrs used for the quantitative realt time RT-PCR.**

| Gene Name | Gene ID | Primer Forward (5’-3’) | Primer Reverse (5’-3’) |
| --- | --- | --- | --- |
| *SlTOM1.1* | *Solyc01g096730* | TCAGACAAGAACTTATGATCGT | TCACCCTGTTTCCCGACTAA |
| *SlTOM1.2* | *Solyc01g096740* | CTGACTTTCAAACCATTCCTTG | CACGTAAAATAGAATATTAAAAAAAT |
| *SlTOM1.3* | *Solyc10g076940* | TACTTCTGACCCTCAGACCC | CACGAGCCTCTCTTGCATT |
| *SlYSL1* | *Solyc08g083060* | CAGTTGCTTCTGGTTTGATCT | TCAAGAAGCCAAGAATTTCATG |
