# Supplementary material for: Single and Combined Fe and S Deficiency Differentially Modulate Root Exudate Composition in Tomato: A Double Strategy for Fe Acquisition?
Source: Int J Mol Sci. 2020 Jun 5;21(11):4038. doi: 10.3390/ijms21114038 (PMC7312093; doi:10.3390/ijms21114038)
Supplement: Supplementary file 1 [file ijms-21-04038-s001.zip › Supplementary Table 2.docx]

**Supplementary Table 2.**  Multivariate Analysis of Variance (MANOVA) table reporting the p-value Bonferroni-corrected of a multivariate comparison evaluating differences in the value of the biomass (root and shoot weight) and the chlorophyll content among the treatments (*i.e.* C=control, F=Fe deficiency, S=S deficiency, D=dual deficiency).

|  | **C** | **F** | **S** | **D** |
| --- | --- | --- | --- | --- |
| **C** | - | < 0.05 | ns | < 0.001 |
| **F** | < 0.05 | - | < 0.05 | < 0.01 |
| **S** | ns | < 0.05 | - | < 0.001 |
| **D** | < 0.001 | < 0.01 | < 0.001 | - |
