# Supplementary material for: Single and Combined Fe and S Deficiency Differentially Modulate Root Exudate Composition in Tomato: A Double Strategy for Fe Acquisition?
Source: Int J Mol Sci. 2020 Jun 5;21(11):4038. doi: 10.3390/ijms21114038 (PMC7312093; doi:10.3390/ijms21114038)
Supplement: Supplementary file 1 [file ijms-21-04038-s001.zip › Supplementary Table 6.docx]

| **Class** | **Compound** | **FC Fe deficiency** | **FC S deficiency** | **FC Fe + S deficiency** |
| --- | --- | --- | --- | --- |
| **Acetoacetates** | acetoacetate | -1.7 | -11 | -8.7 |
| **Acids, Carbocyclic** | diethyl phthalate | 3.3 | -9.5 | -1.3 |
|  | Dihydro-p-coumaric acid | -5.4 | 0.19 | -0.54 |
|  | Methoxyphenylacetic acid | -5.4 | 0.19 | -0.54 |
|  | mono(2-ethylhexyl)phthalate | 15 | 16 | 14 |
| **Alkaloids** | trigonelline | 0.13 | 1 | -17 |
| **Alkanes** | eicosane | -15 | -15 | 1.8 |
| **Alkenes** | 1-octadecene | - | 1.4 | 13 |
| **Amides** | adipamide | 24 | - | 15 |
| **Amino Acids** | L-allothreonine | 4.1 | -3.1 | -8.9 |
|  | Beta- alanine | -0.35 | -7.8 | -8 |
|  | DL-3-aminoisobutyric acid | 19 | 18 | - |
|  | DL-isoleucine | -1.3 | -9.4 | -7.4 |
|  | glycine | 25 | - | - |
|  | L-citrulline | 5.7 | 1.2 | 5.1 |
|  | L-glutamine | 1.5 | 1.1 | -5.6 |
|  | L-homoserine | -1.1 | -10 | -20 |
|  | L-serine | 20 | 19 | 11 |
|  | N-acetyl-ornithine | 19 | - | 11 |
|  | indole 3-propionic acid | -18 | -5.6 | 4.9 |
|  | L-proline | -11 | -11 | -17 |
|  | L-tryptophan | -0.66 | -0.078 | -5.1 |
| **Anisoles** | 3-Methoxyacetophenone | -8.4 | -4.8 | 1.7 |
|  | Anethole | -5.6 | -0.45 | -0.34 |
|  | Estragole | -5.6 | -0.45 | -0.34 |
| **Ascorbic Acid** | L-Ascorbic Acid | -3.4 | -2.3 | 0.51 |
| **Benzaldehydes** | 3-hydroxybenzaldehyde | 0.36 | -5.6 | 0.73 |
|  | 4-hydroxybenzaldehyde | -2.3 | -2.3 | -13 |
|  | benzaldehyde | -0.034 | -0.71 | -5.8 |
|  | Syringaldehyde | 0.16 | -0.35 | -6 |
|  | Vanillin | -11 | -4.9 | -16 |
| **Benzene Derivatives** | 1,2,4,5-tetramethylbenzene | 4.8 | -6.1 | 3.9 |
|  | biphenyl | -15 | -3.4 | -15 |
|  | p-cymene | -0.7 | -5.7 | -1.4 |
| **Benzimidazoles** | 5,6 - dimethylbenzimidazole | -6.6 | -9.2 | -21 |
|  | 5,6 - dimethylbenzimidazole | -20 | -20 | -6.4 |
| **Benzoates** | 4-isopropylbenzoic acid | -3.4 | -12 | 1.2 |
|  | m-toluic acid | 0.24 | -8.5 | -0.11 |
|  | benzoic acid | -4.4 | -16 | 0.31 |
| **Benzodiazepinones** | Flurazepam | -0.23 | -2.2 | -7.1 |
| **Benzothiazoles** | benzothiazole | -5.4 | -2.6 | -4.7 |
| **Benzyl Alcohols** | 2-hydroxybenzyl alcohol | -3.1 | -18 | -8.3 |
|  | 4-isopropylbenzyl alcohol | 2.2 | -6.6 | -7.8 |
|  | benzyl alcohol | -16 | -16 | -7.4 |
| **Butyrates** | gamma-aminobutyric acid | -0.16 | -15 | -20 |
|  | 2-hydroxybutyric acid | 18 | 22 | - |
|  | 4-acetamidobutyric acid | - | 22 | 13 |
|  | L-(+) lactic acid | 1.8 | -14 | -5.3 |
| **Caffeic Acids** | 1,3-Dicaffeoylquinic acid | -6.5 | -6.4 | 0.026 |
|  | 4-Caffeoylquinic acid | 5 | 0.22 | 5.4 |
|  | 5-Caffeoylquinic acid | 5 | 0.22 | 5.4 |
|  | Caffeic acid | -5.8 | -6 | 0.36 |
|  | Dicaffeoylquinic acid | -6.5 | -6.4 | 0.026 |
|  | Dihydrocaffeic acid | 0.16 | -0.35 | -6 |
|  | Hydroxycaffeic acid | -7.5 | -3.5 | -8.2 |
| **Carbamates** | carbamic acid ethyl ester (urethane) | 0.15 | -4.1 | -8.3 |
| **Carotenoids** | Lutein | 5.4 | 0.083 | -0.24 |
|  | Phytofluene | -0.79 | 0.29 | -5.8 |
|  | Zeaxanthin | 5.4 | 0.083 | -0.24 |
| **Ceramides** | DL-dihydrosphingosine | 2 | 2.3 | -19 |
| **Cinnamates** | 1,5-Dicaffeoylquinic acid | -6.5 | -6.4 | 0.026 |
|  | 1-Caffeoylquinic acid | 5 | 0.22 | 5.4 |
|  | 3,4-Dicaffeoylquinic acid | -0.6 | -6.4 | 0.026 |
|  | 3,5-Dicaffeoylquinic acid | -0.062 | 0.017 | 6.5 |
|  | 3-Caffeoylquinic acid | 5 | 0.22 | 5.4 |
|  | Isoferulic acid | 6.5 | 5.7 | 6.2 |
| **Coumaric Acids** | 1,2-Disinapoylgentiobiose | 5.5 | 10 | -5.1 |
|  | 24-Methylcholesterol ferulate | 6.2 | 6.4 | 3.6 |
|  | 4-Vinylguaiacol | -8.4 | -4.8 | 1.7 |
|  | Ferulic acid | 6.5 | 5.7 | 6.2 |
|  | m-Coumaric acid | 0.16 | -0.35 | -6 |
|  | o-Coumaric acid | 0.16 | -0.35 | -6 |
|  | p-Coumaric acid | 0.16 | -0.35 | -6 |
|  | p-Coumaroyl glycolic acid | -0.075 | -0.09 | 16 |
|  | Sinapic acid | -0.88 | -0.53 | -6.3 |
|  | Sitostanyl ferulate | -7.5 | -7.3 | -1.4 |
|  | Sitosterol ferulate | -7.5 | -7.3 | -1.4 |
| **Coumarins** | xanthotoxin | 6.2 | -17 | -4.6 |
|  | 12a-Hydroxy-9-Demethylmunduserone-8-Carboxylic Acid | -0.33 | -8.7 | -9.3 |
|  | 4-Hydroxycoumarin | 0.32 | 6.5 | 6.6 |
|  | dihydrocoumarin 4 | 19 | 19 | - |
|  | Umbelliferone | 0.32 | 6.5 | 6.6 |
| **Cyclitols** | conduritol epoxide | 21 | 11 | 19 |
| **Cyclohexenes** | limonene | -17 | -9.5 | -17 |
| **Deoxyadenosines** | 2', 5'-Dideoxyadenosine | -0.57 | -16 | 1 |
| **Dicarboxylic Acids** | succinic acid | -0.13 | -20 | -20 |
|  | 3-hydroxypropanoic acid | 1.1 | -2.7 | -2.7 |
|  | malonic acid | 25 | 8.3 | 14 |
|  | oxalic acid | 0.6 | -10 | -8.1 |
| **Diterpenes, Abietane** | Carnosic acid | -5.2 | -0.26 | -0.28 |
| **Ethanol** | phenylethyl alcohol | 17 | - | 11 |
| **Ethanolamines** | D-sphingosine | 22 | 13 | - |
|  | ethanolamine | 21 | - | 11 |
| **Flavones** | 3,4',5,6,7-Pentamethoxyflavone | 1.5 | 2.6 | -13 |
|  | Acacetin | -0.16 | -8.9 | -0.34 |
|  | Cirsimaritin | -1 | -1.3 | -2.7 |
|  | Luteolin 6-C-glucoside | -6.2 | 0.082 | 0.1 |
|  | Luteolin 7-O-glucoside | -6.2 | 0.082 | 0.1 |
|  | Quercetin 3-O-rhamnoside | -6.2 | 0.082 | 0.1 |
| **Flavonoids** | 3,7-Dimethoxyflavone | 18 | 8.6 | - |
|  | 2-hydroxychalcone | -0.79 | -19 | -0.82 |
|  | 3-hydroxyflavone | 19 | 8.7 | 14 |
|  | 6-Geranylnaringenin | 4.9 | 10 | 5.2 |
|  | 6-Prenylnaringenin | -7.9 | -0.56 | -0.84 |
|  | 8-Prenylnaringenin | -7.9 | -0.56 | -0.84 |
|  | Isoxanthohumol | 0.46 | 5.9 | 0.32 |
|  | Xanthohumol | 0.46 | 5.9 | 0.32 |
| **Glutamates** | beta-glutamic acid | -4.5 | -16 | -24 |
|  | L-glutamic acid | - | 12 | - |
| **Glycols** | 1,3-propanediol | - | 21 | 12 |
|  | 2,3-butanediol | -0.92 | -14 | -7.8 |
|  | 2-amino-2-methyl-1,3-propanediol | -0.41 | -8.6 | -6.6 |
|  | 2-butyne-1,4-diol | -4.9 | -11 | -3.9 |
| **Hexosamines** | galactosamine | -0.096 | -25 | -25 |
| **Hexoses** | fructose | 7 | -8.8 | -6.4 |
| **Hydantoins** | 1-methylhydantoin | 3 | 2.5 | -2.3 |
| **Hydroxamic Acids** | acetohydroxamic acid | -0.89 | -5.7 | -5.1 |
| **Hydroxybenzoates** | 2-Hydroxybenzoic acid | 1.1 | 0.53 | -11 |
|  | 4-Hydroxybenzoic acid | 1.1 | 0.53 | -16 |
|  | p-anisic acid | -2.8 | -2.6 | -17 |
|  | salicylic acid | -0.18 | 3.2 | 4.1 |
|  | Vanillic acid | -0.075 | -0.09 | 16 |
| **Hydroxyquinolines** | 4-hydroxyquinoline | -4.9 | -8.5 | 1.3 |
| **Imino Acids** | iminodiacetic acid | -26 | -26 | -11 |
|  | N-(2-hydroxyethyl)iminodiacetic acid | 22 | 8.7 | 12 |
| **Isoflavones** | 6''-O-Malonyldaidzin | 7.2 | 8.4 | 8.3 |
|  | Daidzin | 0.16 | -0.061 | -6 |
|  | Formononetin | 17 | 17 | 16 |
| **Isothiocyanates** | benzyl isothiocyanate | 15 | - | - |
| **Kaempferols** | Kaempferol 3-O-glucoside | -6.2 | 0.082 | 0.1 |
|  | Kaempferol 7-O-glucoside | -6.2 | 0.082 | 0.1 |
| **Keto Acids** | 3-methyl-2-oxobutanoic acid | 18 | - | 17 |
| **Ketones** | 2-undecanone | -4.3 | -4.3 | -4.3 |
|  | 4-O-Methylphloracetophenone | -19 | -6.8 | -19 |
|  | 6-methyl-5-hepten-2-one | 0.79 | -7.8 | -15 |
| **Lactates** | 3-phenyllactic acid | 15 | - | 19 |
| **Methamphetamine** | Benzphetamine | 17 | 3.8 | 13 |
| **Nicotinic Acids** | nicotinic acid | 16 | 1.9 | 10 |
| **Nitriles** | 3-aminopropionitrile | 0.95 | -25 | -18 |
| **OH-FA_17_-1_1** | Avocadyne | -0.9 | -14 | -0.9 |
| **Organic Chemicals** | urea | 2.2 | -7 | -9.2 |
| **ortho-Aminobenzoates** | Avenanthramide 2c | 8.4 | 6.3 | 6.2 |
|  | Avenanthramide 2p | 13 | 16 | 16 |
| **Oxaloacetates** | oxalacetic acid | 0 | 3.4 | 15 |
| **Peptides** | L-leucine | -0.55 | -13 | -0.24 |
| **Phenols** | 2,3-dihydroxybiphenyl | -3.2 | 1.4 | -17 |
|  | 2-aminophenol | - | - | 14 |
|  | 3,5-dihydroxyphenylglycine | -2 | -3.2 | -18 |
|  | 2,6-ditert-butyl-4-methylphenol | 20 | 19 | - |
|  | 4-Ethylphenol | 7.1 | 6.4 | 6 |
|  | 4-methylcatechol | -0.25 | -0.7 | -19 |
|  | 4-vinylphenol | 17 | 18 | 13 |
|  | 5-Heneicosenylresorcinol | -5.1 | 0.44 | 0.5 |
|  | orcinol | 1.5 | -7.8 | -16 |
|  | p-Coumaric acid ethyl ester | 0.032 | -5.4 | -6.2 |
|  | Resveratrol | -6.6 | 4.3 | 11 |
|  | Sesamol | 1.1 | 0.53 | -11 |
|  | thymol | -0.42 | -7.5 | -8.1 |
| **Phenylacetates** | 3,4-Dihydroxyphenylacetic acid | -0.075 | -0.09 | 16 |
|  | 4-Hydroxyphenylacetic acid | -11 | -4.9 | -16 |
|  | Homovanillic acid | 0.16 | -0.35 | -6 |
| **Phenylpyruvic Acids** | phenylpyruvate | -3.1 | -5.8 | -3.1 |
| **Pipecolic Acids** | pipecolic acid | -17 | -17 | -5.8 |
| **Polycyclic Aromatic Hydrocarbons** | cis-1,2-dihydro-1,2-naphthalenediol | 18 | 17 | 19 |
|  | 9-hydroxyfluorene | -0.0005 | -3.7 | -8.8 |
|  | fluorene | -3.8 | -4.5 | -11 |
|  | phenanthrene | 13 | 16 | 14 |
| **Polymers** | phosphoric acid | 23 | 8.6 | 13 |
| **Pyridines** | 4-hydroxypyridine | 0 | - | 8 |
| **Saturated FA** | 2-ethylcaproic acid | -0.12 | -5 | 0.11 |
|  | Arachidic acid | -5.9 | -5.6 | 0.43 |
|  | azelaic acid | 19 | 4 | - |
|  | behenic acid | -0.32 | -0.044 | -20 |
|  | Capric acid | 0.29 | -7 | -0.14 |
|  | caprylic acid | 0.058 | -21 | -21 |
|  | Myristic acid | 0.48 | 0.64 | 7.2 |
|  | palmitic acid | - | 17 | 23 |
|  | stearic acid | 0.77 | 6.8 | 18 |
| **Sugar Alcohols** | allo-inositol | -19 | -7.2 | -15 |
|  | diglycerol | 0.93 | -22 | -10 |
|  | glycerol | 0.6 | -16 | -1.2 |
|  | L-dithiothreito | -24 | -11 | -1.6 |
| **Thiazoles** | 4-methyl-5-thiazoleethanol | -1.5 | -17 | 2.7 |
| **Thioglycolates** | thioglycolic acid | - | 15 | 25 |
| **UnSaturated FA** | elaidic acid | 18 | - | - |
| **Valerates** | beta-hydroxyisovalerate | 0.24 | -6.4 | -8.2 |
|  | valeramide | -25 | -11 | -25 |
| **Other compounds** | phytosphingosine | 2.9 | -0.43 | -13 |
|  | 2-Methylene-5-(2,5-Dioxotetrahydrofuran-3-YL)-6-OXO--10, 10-Dimethylbicyclo[7:2:0]Undecane 1 | 18 | 10 | 17 |
|  | benzoxazinone | 15 | 0.02 | -0.059 |
|  | benzyl-desulfoglucosinolate | -11 | -5.1 | -16 |
