# Supplementary figures and images for: Single and Combined Fe and S Deficiency Differentially Modulate Root Exudate Composition in Tomato: A Double Strategy for Fe Acquisition?
Source: Int J Mol Sci. 2020 Jun 5;21(11):4038. doi: 10.3390/ijms21114038 (PMC7312093; doi:10.3390/ijms21114038)

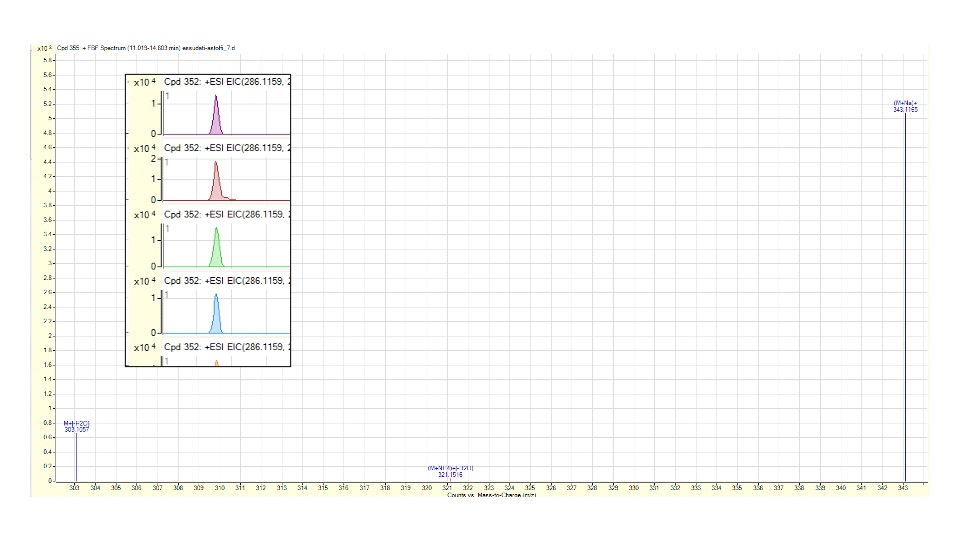

Supplement: Supplementary file 1 [file ijms-21-04038-s001.zip › Supplementary Fig 1.jpg]

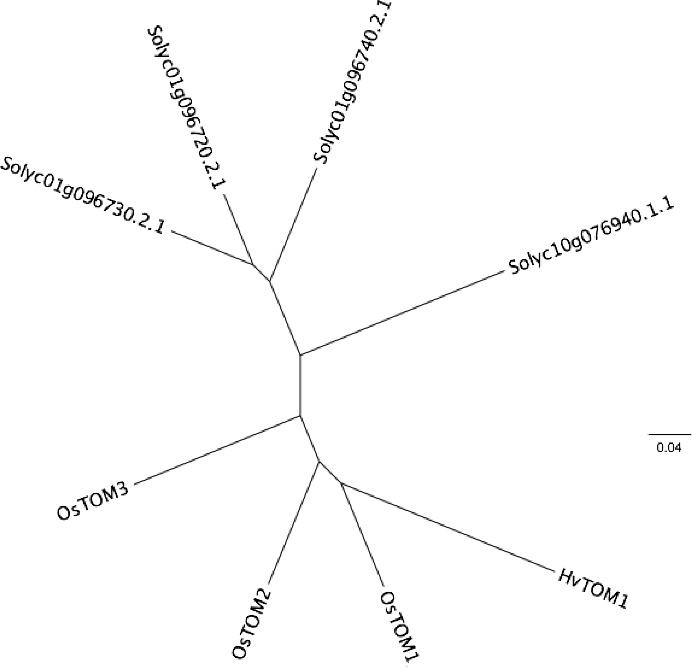

Supplement: Supplementary file 1 [file ijms-21-04038-s001.zip › Supplementary Fig 2.tif]

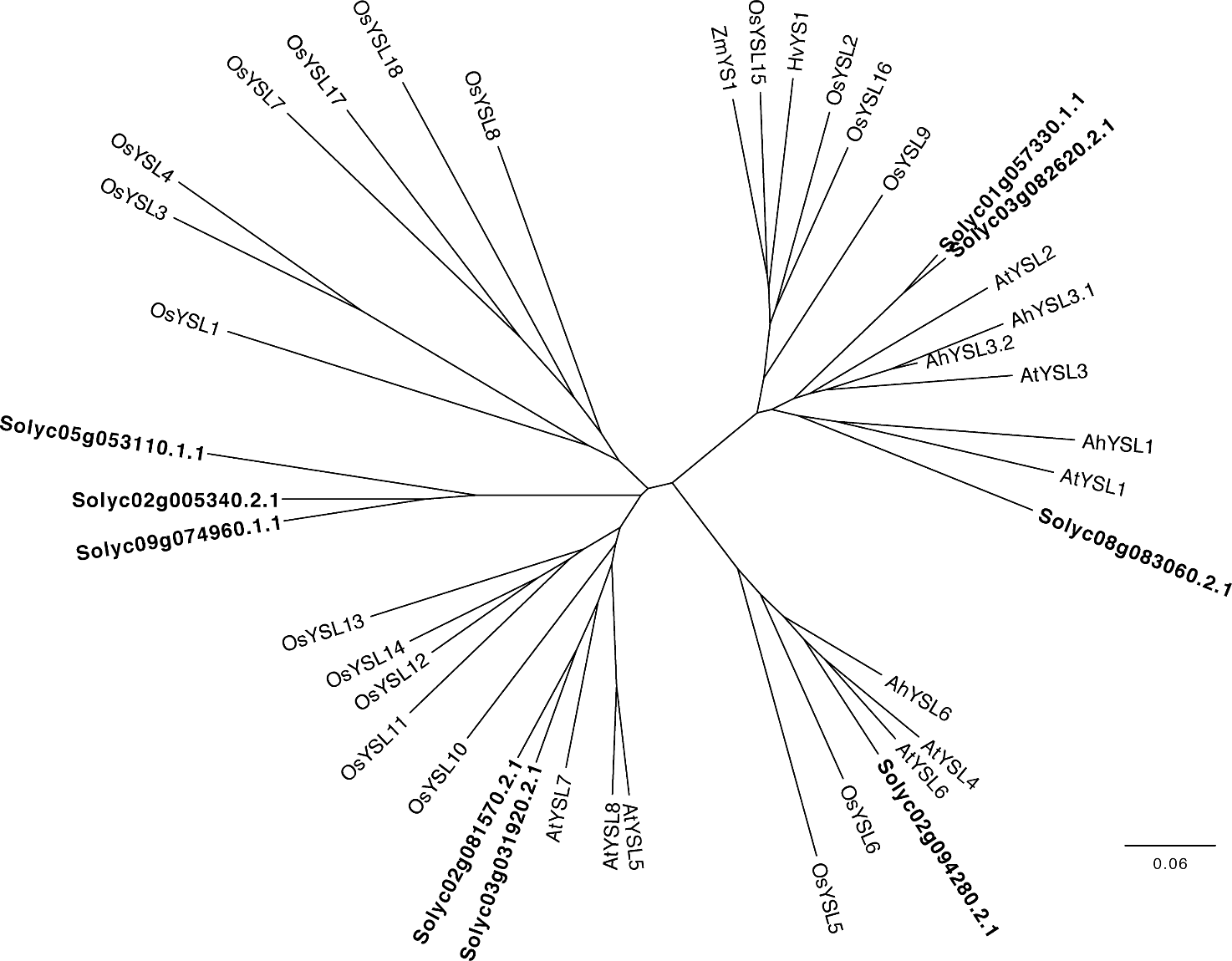

Supplement: Supplementary file 1 [file ijms-21-04038-s001.zip › Supplementary Fig 3.tif]

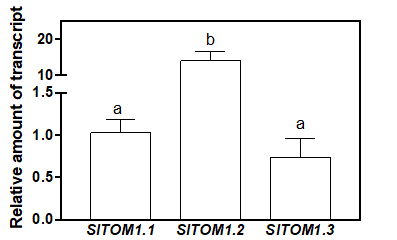

Supplement: Supplementary file 1 [file ijms-21-04038-s001.zip › Supplementary Fig 5.tif]
